# Transcriptional control of axonal guidance and sorting in dorsal interneurons by the Lim-HD proteins Lhx9 and Lhx1

**DOI:** 10.1186/1749-8104-4-21

**Published:** 2009-06-19

**Authors:** Oshri Avraham, Yoav Hadas, Lilach Vald, Sophie Zisman, Adi Schejter, Axel Visel, Avihu Klar

**Affiliations:** 1 Department of Medical Neurobiology, IMRIC, Hebrew University, Hadassah Medical School, Jerusalem, Israel; 2 Genomics Division, Lawrence Berkeley National Laboratory, Berkeley, California 94720, USA

## Abstract

**Background:**

Lim-HD proteins control crucial aspects of neuronal differentiation, including subtype identity and axonal guidance. The Lim-HD proteins Lhx2/9 and Lhx1/5 are expressed in the dorsal spinal interneuron populations dI1 and dI2, respectively. While they are not required for cell fate acquisition, their role in patterning the axonal trajectory of dI1 and dI2 neurons remains incompletely understood.

**Results:**

Using newly identified dI1- and dI2-specific enhancers to trace axonal trajectories originating from these interneurons, we found that each population is subdivided into several distinct groups according to their axonal pathways. dI1 neurons project axons rostrally, either ipsi- or contra-laterally, while dI2 are mostly commissural neurons that project their axons rostrally and caudally. The longitudinal axonal tracks of each neuronal population self-fasciculate to form dI1- and dI2-specific bundles. The dI1 bundles are spatially located ventral relative to dI2 bundles. To examine the functional contribution of Lim-HD proteins to establishment of dI axonal projections, the Lim-HD code of dI neurons was altered by cell-specific ectopic expression. Expression of Lhx1 in dI1 neurons caused a repression of Lhx2/9 and imposed caudal projection to the caudal commissural dI1 neurons. Complementarily, when expressed in dI2 neurons, Lhx9 repressed Lhx1/5 and triggered a bias toward rostral projection in otherwise caudally projecting dI2 neurons, and ventral shift of the longitudinal axonal fascicule.

**Conclusion:**

The Lim-HD proteins Lhx9 and Lhx1 serve as a binary switch in controlling the rostral versus caudal longitudinal turning of the caudal commissural axons. Lhx1 determines caudal turning and Lhx9 triggers rostral turning.

## Background

The diverse functions of the vertebrate nervous system depend on synaptic connections between specific classes of neurons and their targets. Neurons differ from each other by their type of afferent input, cell body positioning along the body axis, axonal trajectory and axonal target. The projection of axons to their targets occurs in a stepwise manner, under the control of guidance cues arrayed at discrete locations along the pathway of axonal growth. A specific axonal pathway of a neuron, governed by a transcriptional code, is manifested by the expression of receptors for guidance molecules that interpret the guidance cues *en route *and at their putative target [1,2].

In vertebrates, the coordinated development of neurons and their targets has been well documented in the context of the peripheral projections of spinal motor neurons. Motor neurons innervate many different muscle targets, and the location of motor neurons within the spinal cord is linked to target position. Lim-HD proteins control aspects of neuronal differentiation, such as subtype identity and axonal guidance (reviewed in [3]). The broad repertoire of specification by Lim-HD factors is exemplified in the development of motor neurons [4-9]. While the early expression of Isl1 is required for the differentiation of all the motor neurons [6], later in development, Isl1 confers LMCm subtype identity to motor neurons and directs LMCm axons to the ventral limb. In a contrasting and complementary manner, Lhx1 confers LMCl subtype identity and directs LMCl axons to the dorsal limb [4,5].

The uncertainty about the role of Lim-HD proteins in the control of motor axon pathfinding stems from the fact that many genes of this class control earlier developmental decisions – the regulation of neural pattern, cell specification, and cell survival [10]. A replacement of the Lim-HD code of LMC neurons, via ectopic expression of Isl1 or Lhx1, causes a binary switch in cell fate, where ectopic Isl1-expressing motor neurons adopt LMCm subtype identity, and ectopic Lhx1-expressing motor neurons become LMCl neurons [4]. Similarly, the LIM homeobox genes *Lhx3 *(*Lim3*) and *Lhx4 *(*Gsh4*) are transiently expressed by spinal motor neurons but appear to specify neuronal subtype identity and migratory behaviour, indirectly influencing the position at which motor axons emerge from the spinal cord [7]. Nevertheless, studies in *Drosophila *have shown that Lim-HD proteins direct motor axon projections without influencing neuronal fate [11,12], suggesting that some of their vertebrate counterparts may have similar roles.

Spinal sensory neurons are derived from several populations of dorsal interneurons (dI1-6) in the embryonic dorsal spinal cord that are distinguished by a transcriptional code and differentiated cell body positions. dI1-3 neurons differentiate from distinct groups of ventricular zone progenitor cells that express the basic helix loop helix (bHLH) transcription factors Atoh1, Ngn1/2 or Mash1, respectively. As the dI1-3 neurons differentiate, Lim-HD transcription factors are expressed: Lhx2 and Lhx9 in dI1, Lhx1 and Lhx5 in dI2, and Isl1 in dI3 [13,14]. Gene targeting and transgenesis in mice have revealed that dI1 neurons project their axons ipsi- and contra-laterally toward the brain [15,16], and dI2 neurons project their axons contra-laterally [17]. However, the precise *en route *axonal pathway, as well the topographic organization of dI axons within the neural tube, is not known.

In this study we used genetic assays in chick embryos to address the basis of the selection of interneuron axonal trajectory within the developing neural tube. Initially, taking advantage of novel enhancer elements, we mapped the axonal trajectories of dI1 and dI2 neurons. Each dI has a unique pattern of axonal projections. dI1 neurons project their axons rostrally along two pathways: either ipsi- or contra-laterally. dI2 are mostly commissural neurons. After crossing the floor plate the rostral dI2 axons turn rostrally, while the caudal dI2 axons turn caudally. To begin to understand the possible role of Lim-HD in patterning the axonal trajectories of spinal interneurons, the Lim-HD code of dI1 and dI2 neurons was altered by cell type-specific ectopic expression. We found that Lhx1, ectopically expressed in dI1 neurons, confers caudal projection to the otherwise rostrally projecting commissural dI1 axons; while Lhx9, expressed in dI2 neurons, causes a rostral bias to the caudally projecting dI2 axons. Thus, Lim-HD proteins control the longitudinal axonal choice of dI1 and dI2 neurons.

## Results

### Enhancer elements

Employment of enhancer elements to drive expression of reporter genes in neurons is a widely used paradigm for tracking axonal projection. For tracking axonal projection of spinal interneurons in vertebrates, germ line-targeted reporter genes yield bilaterally symmetric labelling [15,17,18]. Therefore, it is hard to distinguish between the ipsi- and contra-laterally projecting axons. Unilateral electroporation into the chick neural tube provides a useful means to restrict expression of a reporter gene to one side of the central nervous system, and to follow axonal projection on both sides [19,20]. Mouse enhancer elements are appropriately active in the chick neural tube. Thus, Atoh1, HB9, and HoxA1 enhancer elements drive expression in dI1, motor neurons and floor plate cells, respectively [20-23].

**Figure 1:**
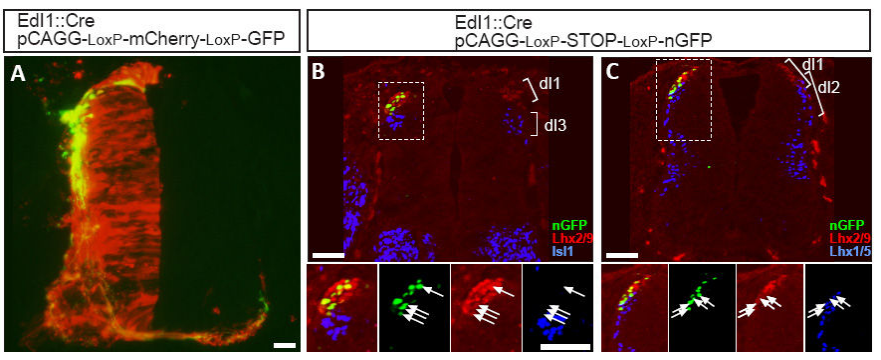
**Characterization of the dI1 enhancer**. The EdI1 enhancer element was cloned upstream of Cre recombinase and electroporated with **(A) **a conditional alternating mCherry/GFP (CAGG-loxP-mCherry-loxP-GFP) or **(B, C) **a conditional nuclear GFP (CAGG-loxP-STOP-loxP-nGFP) plasmid. Chick embryos were electroporated at stage 16 and fixed at stage 23 (B, C) or stage 26 (A). Cross-sections of electroporated neural tube were stained with dI-specific antibodies (B, C). (A) The use of the alternating mCherry/GFP allows the simultaneous detection of the electroporated cells (expressing mCherry) and cells that express the EdI1 enhancer (expressing GFP). Most of the cells, along the entire dorsal/ventral levels, express mCherry, while a subpopulation of dorsally located cells expresses GFP. (B) Cross-sections of electroporated neural tube were stained with Isl1 and Lhx2/9 antibodies. Nuclear GFP (nGFP)-expressing neurons are Lhx2/9^+^/Isl1^-^. (C) Cross-sections of electroporated neural tube were stained with Isl1 and Lhx1/5 antibodies. nGFP expressing neurons are Lhx2/9^+^/Lhx1/5^-^. The boxed areas in (B, C) are represented as enlargements in their different channels at the bottom of each panel. The arrows point to the nGFP-expressing cells. Scale bars: 50 μm.

**Figure 2:**
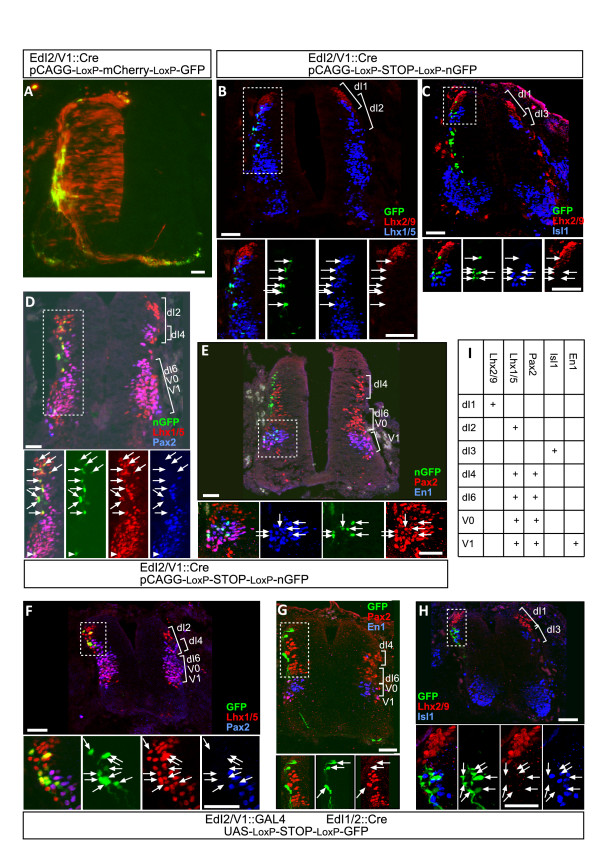
**Characterization of dI2 enhancers**. The EdI2/V1 and EdI1/2 enhancer elements were cloned upstream of Cre recombinase or Gal4 and electroporated with **(A) **a conditional alternating mCherry/GFP plasmid (CAGG-loxP-mCherry-loxP-GFP), **(B-E) **a conditional nuclear GFP (nGFP) plasmid (CAGG-loxP-STOP-loxP-nGFP) and **(F-H) **a double conditional GFP plasmid (UAS-loxP-STOP-loxP-GFP). Chick embryos were electroporated at stage 16 and fixed at stage 23 (B-H) or stage 26 (A). (A) Embryos were co-electroporated with a EdI2/V1::Cre plasmid and a conditional alternating mCherry/GFP plasmid. Most of the cells, along the entire dorsal/ventral levels, express mCherry, while a subpopulation of dorsally and medial-laterally located cells expresses GFP. (B-E) Embryos were co-electroporated with a EdI2/V1::Cre plasmid and a conditional nGFP plasmid. Cross-sections of electroporated neural tube were stained with cell fate markers. nGFP-expressing neurons at the dorsal neural tube are dI2 neurons, as indicated by the expression of Lhx1/5^+^/Lhx2/9^- ^(B), Lhx2/9^-^/Isl1^- ^(C), and Lhx1/5^+^/Pax2^- ^(D); at the medial neural tube, nGFP-expressing neurons are V1 neurons, as indicated by the co-expression of Lhx1/5^+^/Pax2^+ ^(arrowhead in (D)) and En1^+^/Pax2^+ ^(E). (F-H) Expression of GFP in neurons that co-express the EdI2/V1 and EdI1/2 enhancers. Embryos were electroporated with three plasmids: EdI2/V1::Gal4, EdI1/2::Cre and the double conditional GFP plasmid. Note that GFP is expressed in the cytoplasm, axons and dendrites. The arrows point to the center of the neurons. GFP-expressing neurons are Lhx1/5^+^/Pax^- ^(F), Pax^-^/En1^- ^(G) and Lhx2/9^-^/Isl1^- ^(H). (I) A table showing the specificity of the antibodies used in Figures 1 and 2. Boxed areas in the panels are represented as enlargements in their different channels at the bottom of each. The arrows point to the nGFP- and GFP-expressing cells. Scale bars: 50 μm.

Large-scale transgenic mouse screens of highly conserved non-coding sequences in the human genome have revealed several hundred enhancer elements that target β-galactosidase reporter gene expression to specific developmental structures and cell types in transgenic mice at embryonic day (E)11.5 [24-26]. Two enhancer elements, seemingly expressed in dI neurons, were further analyzed employing *in ovo *electroporation: #284, located between the *Pou3f2 *gene and the *C6orf167 *open reading frame on human chromosome 6; and #169, located between *Foxd3 *and *Atg4c *genes on human chromosome 1. The mouse #284 and #169 elements were cloned upstream to Cre recombinase. To verify the specificity to dorsal interneurons, the Enhancers::Cre plasmids were electroporated into stage 16 to 17 chick hemi-tube along with a Cre-dependent mCherry/green fluorescent protein (GFP) plasmid [pCAGG-LoxP-mCherry-LoxP-GFP], which enables the simultaneous detection of the electroporated cells (expressing mCherry) and the enhancer-expressing cells (expressing GFP) (for more details, see Materials and methods). GFP expression is restricted to dorsal neurons (#284; Figure 1A) or the medial lateral neurons (#169, Figure 2A), while mCherry is expressed along the entire ventral/dorsal aspect of the electroporated hemi-tube (Figures 1A and 2A).

To further characterize the cell type specificity of the enhancers, the embryos were co-electroporated along with a Cre-dependent nuclear GFP (nGFP). To determine the identity of reporter-expressing cells, embryos were analyzed at stage 23 to 24 by co-staining with dI-specific antibodies to Lhx2/9 (dI1), Lhx1/5 (dI2, dI4, dI6, V0, V1), Isl1 (dI3), Pax2 (dI4, dI6, V0, V1) and Engrailed1 (V1) (Figure 2I). nGFP expression under the control of #284 is restricted to dI1 neurons, as indicated by co-staining with Lhx2/9 Ab (Figure 1B, C) and the segregation from Lhx1- (Figure 1C) and Isl1-positive neurons (Figure 1B). Of the nGFP-positive neurons, 95.7% (n = 139) are Lhx2/9, and 4.3% (6 of 139) were nGFP-positive but negative to all the above interneuron markers. This minor population may represent progenitors of dI1 that have not upregulated the expression of Lhx2 and Lhx9 yet. The #284 enhancer is herein indicated as EdI1.

Of the neurons that express nGFP under the control of #169, 56% (n = 166) are dI2, as indicated by the co-localization to the dorsal Lhx1/5^+ ^(Figure 2B) and Lhx1/5^+^/Pax2^- ^cells (Figure 2D) and the segregation from Lhx2/9^+ ^and Isl1^+ ^neurons (Figure 2B, C); 35.5% are V1 neurons as indicated by the localization to the medial Pax^+^/En1^+^/Lhx1/5^+ ^neurons (Figure 2D, E). Of the nGFP neurons, 8.5% are presumed to be progenitors of dI2 and V1, since no expression of any cell fate marker was scored. Its proximity to the dI2/V1-specific gene *Foxd3 *suggests that #169 is a dI2/V1-specific enhancer of Foxd3 (herein indicated as EdI2/V1).

For further focusing on the axonal projection pattern of dI2 neurons, the dI2-specific enhancer element (13G) of the *Ngn1 *gene was studied in the chick neural tube [27], utilizing the Cre-dependent nGFP system. Expression of nGFP was detected in dorsal/lateral interneurons that express either Lhx2/9 or Lhx1/5 (Additional file 1). Numerous dorsal interneurons located between the ventricular and marginal zones express nGFP. These neurons are presumed to be progenitors of dI1 and dI2 neurons. The expression of Ngn1 in progenitor neurons supports this assumption. The leakage in dI1 neurons while using the Ngn1-13G enhancer [27], versus the entire Ngn1 enhancer [17], suggests that *cis *elements that are required for repression of expression in dI1 neurons are absent in the 13G enhancer. Hence, in the chick, the Ngn1 13G enhancer is a dI1/2-specific enhancer (herein indicated as Ed1/2).

### Axonal projection of dI1 axons

dI1 neurons give rise to two subpopulations that differ in their cell position, axonal projection and transcription of the Lim-HD proteins: the dI1_comm _population, located at the dorsal neural tube and more ventral/medially, which projects axons toward and across the floor plate; and the dI1_ipsi _population, located in a ventral/lateral position, which projects axons ipsi-laterally. The division into two subpopulations is also evident in the transcription of the Lim-HD proteins Lhx2 and Lhx9. dI1_comm _neurons express Lhx2_high _and Lhx9_low_, while dI1_ipsi _neurons express Lhx9 [16,28].

**Figure 3:**
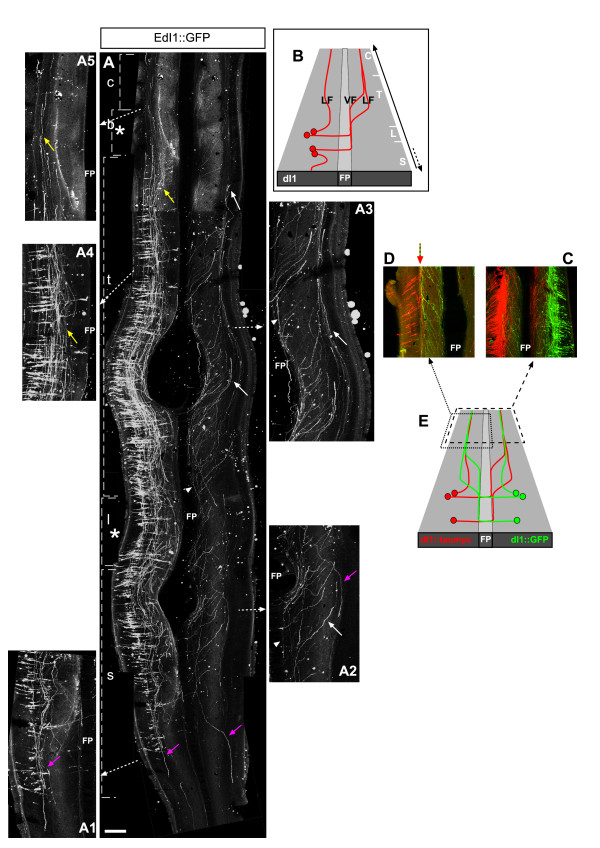
**Axonal projection pattern of dI1 neurons**. **(A) **Chick embryos were electroporated at stage 16 (left side) with EdI1 enhancer along with a Cre-dependent GFP plasmid (EdI1::Cre + pCAGG-LoxP-STOP-LoxP-GFP). At E6, spinal cords were removed, fixed and analyzed as an open-book preparation. Whole neural tubes (sacral to cervical) are presented. Confocal images were taken and photomerged utilizing Photoshop software. **(B) **The schematic illustrates the axonal projection pattern of the dI1 neuronal population. Rostral is up in the image and the schematics. Asterisks represent the levels of the limbs. dI1 neurons have two main rostral axonal projection pathways (A): ipsi-lateral (A4, A5, yellow arrows), and contra-lateral (A, A2, A3, white arrows and arrowheads). After crossing the floor plate (FP), dI1_comm _neurons elongate along the floor plate at the ventral funiculus (VF; white arrowheads) for several segments and, subsequently, turn toward the lateral funiculus (LF; white arrows). dI1_ipsi _axons turn longitudinally and rostrally at the LF of the lumbar, thoracic and cervical levels (A, A4, A5). At the caudal sacral level dI1_ipsi _axons project caudally (A, A1, magenta arrows). **(C, D) **The relative position of dI1_ipsi _and dI1_comm _fascicules at the LF was studied following differential labelling of dI1_ipsi _and dI1_comm _axons. At the LF, dI1_ipsi _and dI1_comm _form one fascicle (green+red arrow in (D)). **(E) **Schematic with boxed areas representing the frames of (C, D). Only the rostrally turning axons are illustrated in (E). c, cervical level; b, brachial level; FP, floor plate; l, lumbar level, s, sacral level; t, thoracic level. Scale bars: 150 μm (A, D); 100 μm (A1–A5); 300 μm (C).

The axonal projection pattern of dI1 neurons within the neural tube was studied at E6 utilizing an open-book preparation of electroporated neural tubes. dI1 neurons, labeled with GFP under the control of EdI1 enhancer, project their axons ipsi- and contra-laterally (Figure 3A, B). The neural tubes of ten embryos were analyzed and yielded similar axonal patterns (Table 1). The contra-laterally projecting axons cross the floor plate, turn rostrally and elongate along the floor plate at the ventral funiculus (VF) for a few segments. They are subsequently deflected diagonally and laterally away from the floor plate (Figure 3A, A2, A3). At the lateral funiculus (LF), dI1_comm _axons turn rostrally whilst converging to a longitudinal bundle. At the cervical level, a longitudinal fascicule is evidenced only in the LF. All the axons at the contra-lateral VF turn toward the LF. dI1_ipsi _axons turn rostrally at the LF (Figure 3A, A4, A5). At sacral levels, few caudally projecting axons are visible (Figure 3A, A1, A3, magenta arrows). The number of caudally versus rostrally projecting axons at the sacral level on the contra-lateral side was scored. 12.5 ± 4.4% (n = 6) of the axons turned caudally. However, it cannot be excluded that beyond E6, more sacral dI1 neurons also project their axons caudally or, alternatively, are eliminated.

**Table 1:** Summary of the axonal phenotypes.

			dI2 crisscross					
						
	dI1 axonal patterning	dI2 commissural axonal patterning	In S	In T	NO	C to R	dI1+dI2 in LF	Number of embryos
EdI1::GFP								
EdI1::taumyc	2/2							2
EdI2/V1::GFP		6/6		6/6				6
EdI2/V1::taumyc		2/2		2/2				2
EdI1/2::GFP		4/4		4/4				4
dI2 only (intersection)		4/4		4/4				4
EdI1::Lim1+taumyc		3/3		3/3				3
EdI2/V1::Lhx9+GFP			4/6	0/6	2/6	5/6		6
EdI2/V1::Lhx2+taumyc		4/4	4/4			4/4		4
EdI2/V1::Lhx9+GFP EdI2/V1::Lhx2+taumyc			1/1			1/1		1
EdI1::taumyc EdI2/V1::GFP							0/2	2
EdI1::taumyc EdI2/V1::Lhx9+GFP							2/2	2

S, sacral; T, thorax; NO, no crisscross; C to R, shift from caudal to rostral projection. dI1+dI2 in LF, co-fasiculation of dI1 and dI2 axons at the lateral funiculus.

The positions of the longitudinal dI1_ipsi _and dI1_comm _fascicules at the LF along the dorsal/ventral axis appear similar. Hence, it is conceivable that dI1_comm _axons from one side of the neural tube, and dI1_ipsi _axons from the other side, fasciculate together. To test this hypothesis, GFP or taumyc were expressed in the two halves of the neural tube, respectively (see Materials and methods). Projection of dI1_comm _GFP-positive axons toward the dI1_ipsi _taumyc axons was inspected (Figure 3C, D, E). dI1_comm _axons turned diagonally toward the dI1_ipsi _bundle. As they contacted the dI1_ipsi _bundle, dI1_comm _axons fasciculated with dI1_ipsi _and turned rostrally (Figure 3D). Thus, homophilic interaction between dI1_comm _and dI1_ipsi _may facilitate axonal turning of dI1_comm _at the LF.

### Axonal projection of dI2 neurons

The Ngn1 enhancer was utilized previously for labelling the axons of dI2 neurons in transgenic mice. Cross-sections and open-book preparation demonstrated that dI2 neurons project their axons toward and across the floor plate [17,20,27]. However, the bi-symmetrical expression of the reporter gene precluded detailed mapping of dI2 axonal trajectories.

The axonal cues of dI2 axons were studied utilizing three paradigms: the EdI2/V1 enhancer – V1 neurons project their axons only ipsi-laterally [29,30] and, thus, the EdI2/V1 enhancer can be used for studying the contra-lateral projection pattern of dI2 neurons (six embryos; Table 1); the Ed1/2 enhancer – divergence from the dI1 axonal pattern, when employing the dI1/2 enhancer, can be attributed to dI2 neurons (four embryos; Table 1); and molecular intersection of the EdI2/V1 and EdI1/2 enhancers – we have designed a method that enables labelling of neurons that co-express the above enhancers (four embryos; Table 1).

**Figure 4:**
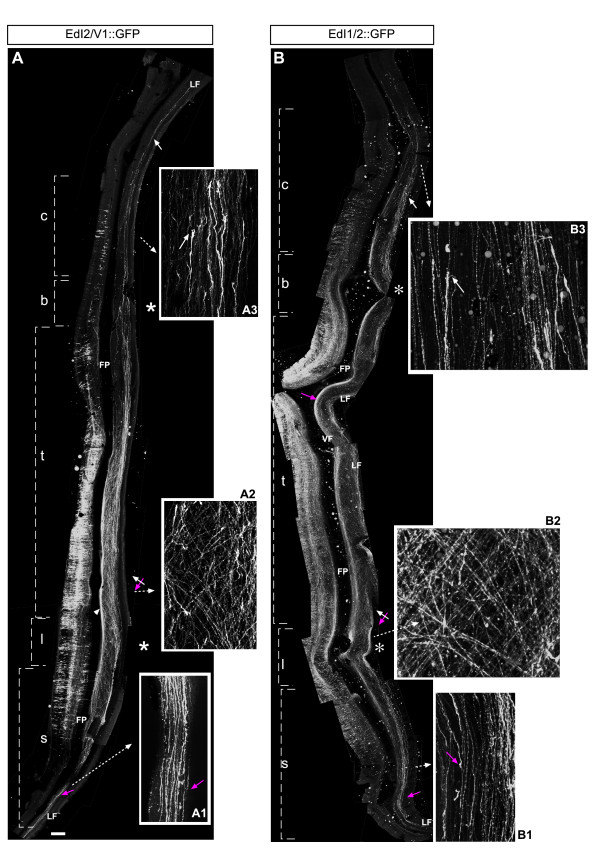
**Axonal projection pattern of contra-laterally projecting dI2 neurons**. Chick embryos were electroporated at stage 16 (left side) with **(A) **EdI2/V1 and **(B) **EdI1/2 enhancers along with a Cre-dependent GFP plasmid (EdI2/V1::Cre or EdI1/2::Cre + pCAGG-LoxP-STOP-LoxP-GFP). At E6, spinal cords were removed, fixed and analyzed as an open-book preparation. Whole neural tubes (sacral to cervical) are presented. Confocal images were taken and photomerged utilizing Photoshop software. Rostral is up in the image and the schematics. At the sacral level at the contra-lateral side dI2_caud _axons turn caudally (A, A1, B, B1, magenta arrows); at the caudal thoracic level axons turn either caudally or rostrally, forming a crisscross pattern (A, A2, B, B2, white and magenta crossed arrows). At the rostral two-thirds of the thoracic level, the brachial and cervical levels dI2_rost _axons turn rostrally (A, A3, B, B3, white arrows). At the sacral and cervical levels axons turn from the ventral funiculus (VF) to the lateral funiculus (LF) (A, A1, A3, B). c, cervical level; b, brachial level; FP, floor plate; l, lumbar level, s, sacral level; t, thoracic level. Asterisks represent the level of the limbs. Scale bar: 200 μm (A); 50 μm (A1–A3); 150 μm (B); 30 μm (B1–B3).

Expression of GFP unilaterally in the chick neural tube under the control of EdI2/V1 revealed that dI2 neurons have two different axonal projection patterns at the contra-lateral side (Figure 4A, Table 1). At the rostral two-thirds of the thoracic level and the brachial and cervical levels, dI2 axons grow toward and across the floor plate. At the contra-lateral side of the floor plate, axons turn rostrally (dI2_rost_; Figure 4A, A3). As with dI1_comm _axons, dI2_rost _axons elongate along the floor plate for a few segments, and subsequently turn laterally and diagonally in the white matter and fasciculate at the LF (Figure 4A). Caudal to the hindlimb, at the lumbar and sacral levels, dI2 axons turn caudally in a mirror-image pattern to the rostrally projecting axons (dI2_caud_). Specifically, they grow ventrally toward the floor plate and turn caudally at the contra-lateral side of it (Figure 4A, A1). Then, they turn laterally and form a dI2_caud _fascicle at the contra-LF. Along the caudal third of the thoracic level, a mixture of caudally and rostrally projecting axons that form a crisscross pattern at the contra-lateral side is evident (Figure 4A, A2). Similar contra-lateral axonal pathways were observed when employing the dI1/2 enhancer (Figure 4B): rostral (Figure 4B, B3), crisscross (Figure 4B, B2) and caudal (Figure 4B, B1). Since dI1 neurons do not project caudally, the caudal projection that is seen utilizing the dI1/2 enhancer is attributed to dI2 neurons.

The co-expression in dI2 plus V1 neurons or dI2 plus dI1 utilizing the EdI2/V1 and EdI1/2 enhancers, respectively, precludes the identification of ipsi-laterally projecting axons. For labelling dI2 neurons solely, an enhancer intersection technique was adopted. The EdI2/V1 and EdI1/2 enhancers are not exclusive to dI2 neurons; however, their intersection occurs in dI2 neurons. In order to label neurons that co-express EdI2/V1 and EdI1/2 enhancers, we combined the Cre/LoxP and the Gal4/UAS systems. Cre was expressed under the EdI1/2 enhancer, and Gal4 under the EdI2/V1 enhancer. The reporter plasmid contains GFP under a dual control of Gal4 and Cre (UAS-LoxP-STOP-LoxP-GFP; for more details, see Materials and methods). GFP-expressing neurons in which the intersection of EdI1/2 and EdI2/V1 enhancers occurs are Lhx1/5^+^/Pax2^- ^(100%, n = 22; Figure 2F), Pax2^-^/En1^- ^(100%, n = 18; Figure 2G) and Lhx2/9^-^/Isl1^- ^(100%, n = 29; Figure 2H). Hence, they are dI2 neurons.

**Figure 5:**
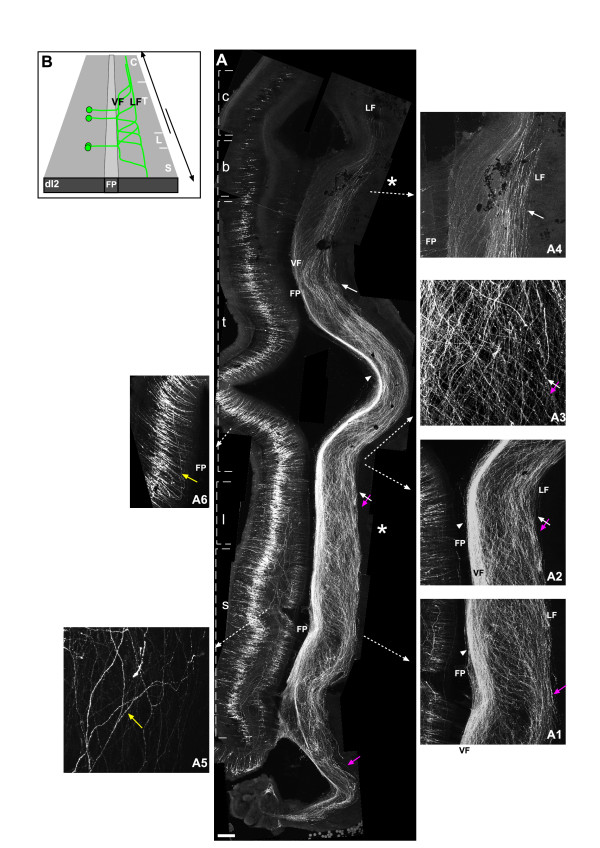
**Axonal projection pattern of dI2 neurons**. **(A, B) **Chick embryos were electroporated at stage 16 with three plasmids: dI1/2::Cre, dI2/V1::Gal4 and UAS::LoxP-STOP-LoxP-GFP. The schematic (B) illustrates the axonal projection pattern of the dI2 neuronal population. At the sacral and lumbar levels on the contra-lateral side, dI2_caud _axons turn caudally (A, A1, magenta arrows). At the caudal thoracic level of the contra-lateral side a crisscross pattern of axons turning either rostrally or caudally is apparent (A, A2, A3, white and magenta crossed arrows). At the rostral thoracic level, and the cervical level, dI2_rost _axons turn rostrally (A, A4, white arrows). Along the entire longitudinal axis, dI2 axons initially form a fascicle at the contra-lateral ventral funiculus (VF; arrowheads in A, A1, A2) and subsequently axons are deflected to the lateral funiculus (LF; A, A4). At the ipsi-lateral side only few axons project longitudinally (A5, yellow arrow). Most of the axons grow toward the floor plate (A, A6). c, cervical level; b, brachial level; FP, floor plate; l, lumbar level, s, sacral level; t, thoracic level. Asterisks represent the level of the limbs. Scale bar: 150 μm (A); 75 μm (A1, A2, A4, A6); 35 μm (A3, A5).

The axonal pathways of dI2 neurons at the ipsi- and contra-lateral sides were studied using E6 open book preparations (Figure 5). The axonal patterning of the commissural dI2 axons, labeled exclusively by the intersection technique, is similar to the pattern observed with EdI2/V1 and EdI1/2 enhancers (Figure 4). Namely, dI2_rost _axons turn rostrally from the rostral two-thirds of the thoracic level (Figure 5A, A4) and either rostrally or caudally at the caudal third of the thoracic level (Figure 5A, A2, A3), and dI2_caud _axons turn caudally from the hindlimb level (Figure 5A, A1). It is difficult to estimate the extent of caudal versus rostral turning at each level due to the axonal abundance of dI2 neurons at the contra-lateral side. However, an inspection of several neural tubes shows that the vast majority of the neurons at the cervical level are dI2_rost _(Additional file 2A) and at the sacral level are dI2_caud _(Additional file 2B).

At the ipsi-lateral side, few longitudinally projecting axons are seen (Figure 5A, yellow arrow in A5; Additional file 2A). The majority of the axons project circumferentially toward the floor plate (Figure 5A, A6; Additional file 3). No longitudinal tracks are observed at either the VF or the LF. To estimate the ratio between the ipsi- and contra-lateral axonal choice of dI2 neurons, the extent of ipsi-/contra-lateral axons at the cervical level was scored. At this level, no cell bodies were labeled on the ipsi-lateral side. Only in one neural tube (n = 4) were longitudinally projecting axons visible at the cervical ipsi-lateral side (Additional file 3). The ratio between ipsi- to contra-lateral axons is 8.6% in that neural tube. Hence, dI2 neurons have mainly commissural axons that elongate longitudinally either rostrally or caudally, depending on their position along the longitudinal axis.

### Lim-HD cross-repression

**Figure 6:**
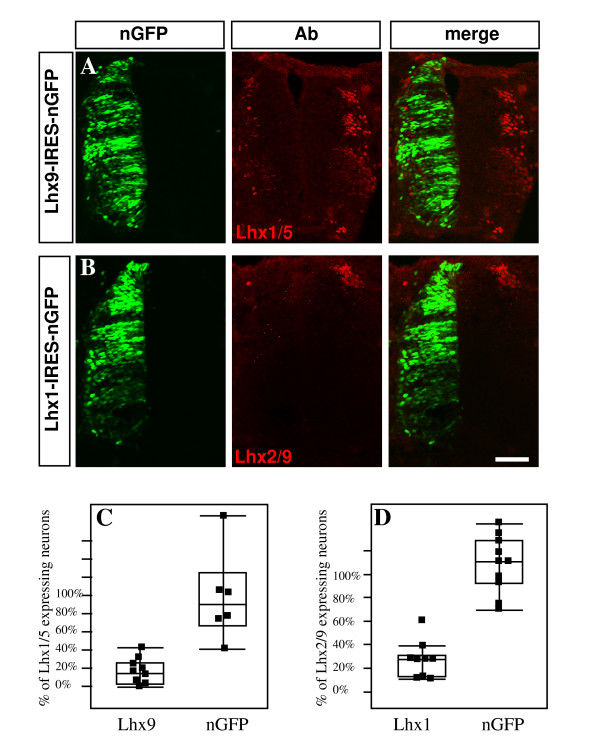
**Lhx9 and Lhx1 cross-repress each other**. **(A, C) **Lhx9 and **(B, D) **Lhx1, cloned in pCIG plasmid, were electroporated at stage 19 into the chick neural tube. At E4, embryos were fixed and stained with antibodies (Ab) to the cognate Lim-HD proteins. A vast reduction in Lhx1/5 (A) is evident after Lhx9 ectopic expression. Lhx1 ectopic expression resulted in a reduction of Lhx2/9 (B). For quantification (C, D), the ratio between dIx-specific neurons expressing ectopic Lim-HD (Lim^ecto^) plus their own Lim-HD (Lim^x^) and the total number of electroporated dIx neurons is presented. Neurons co expressing Lim^ecto ^and Lim^x ^are nuclear GFP (nGFP)+/Lim^x^+. For estimating the total number of electroporated dIx neurons, the number of nGFP-/Lim^x^+ neurons at the electroporated side was subtracted from the number of Lim^x^+ neurons at the control side. The quotient plus the number of nGFP+/Lim^x^+ equals the number of electroporated dIx neurons. Scale bar: 200 μm.

In motor neurons, reciprocal cross-repression between Isl1 and Lhx1 ensures a sharp boundary between the LMC subpopulations [4]. The distinct cell boundaries (Figures 1 and 2) suggest that similar Lim-HD mechanisms may account for dI subdifferentiation. To test whether the Lim-HD code of dI1 and dI2 neurons is maintained through cross-repression, each Lim-HD was expressed uniformly at stage 19 in the chick hemitube (Figure 6). The ratio of neurons co-expressing the ectopic Lim-HD protein and the endogenous Lim-HD protein of the reciprocal dI neurons among the electroporated neurons was measured. In neural tubes electroporated with nGFP, 96% of the electroporated dI1 neurons co-expressed nGFP and Lhx2/9 and 98% of dI2 neurons co-expressed nGFP and Lhx1 (Figure 6C, D). Ectopic expression of Lhx9 resulted in substantial reduction of neurons co-expressing Lhx9 and Lhx1 (Figure 6A). Only 15.76% co-expressed Lhx9 and Lhx1 (Figure 6C). Likewise, Lhx1 affected a comparable decrease in the expression of Lhx9 proteins (Figure 6B). Ectopic Lhx1 resulted in 16.7% of neurons co-expressing Lhx1 together with Lhx9 (Figure 6D).

**Figure 7:**
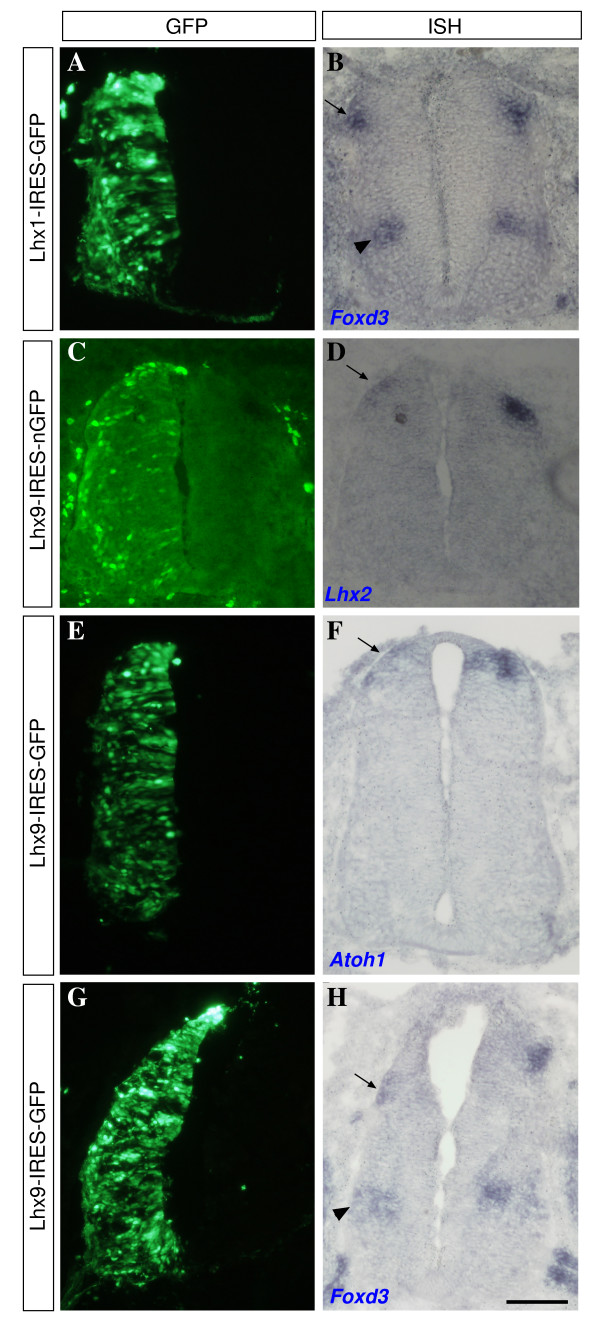
**Lhx9 and Lhx1 do not change dI cell fate**. *In situ *hybridizations with dI-specific genes *Foxd3 *(dI2), and *Lhx2 *and *Atoh1 *(dI1) were preformed on sections of embryos electroporated with **(A, B) **Lhx1-IRES-GFP, **(C, D) **Lhx9-IRES-nGFP and **(E-H) **Lhx9-IRES-GFP. Adjacent sections were used for GFP detection (A, E, G) and for *in situ *hybridization (B, F, H). Alternatively, GFP detection by antibody staining and *in situ *hybridization were performed on the same slide (C, D). Ectopic expression of Lhx1 does not activate the expression of *FoxD3 *(A, B). Ectopic expression of Lhx9 down-regulates the expression of Lhx2 (C, D) and Atoh1 (E, F) and does not affect Foxd3 expression (G, H). Arrows point to dI2 neurons (B, H) and dI1 neurons (D, F). Arrowheads point to V1 neurons (B, H). Scale bar: 150 μm.

Cross-repression may arise from a change of cell fate. Thus, ectopic expression of Lhx9 or Lhx1 may determine dI1 and dI2 fate, respectively, which, as a consequence, will lead to down-regulation of the reciprocal Lim-HD protein. However, the competence of repression at a relatively late stage (stage 19) suggests that cross-repression can be mediated in post-mitotic cells without affecting cell fate. The expression of dI1/2 cell fate markers was studied following ectopic expression of Lhx9 and Lhx1. dI2 neurons express Foxd3. The expression of Foxd3 was not up-regulated following Lhx1 ectopic expression (Figure 7A, B). Thus, Lhx1 is not sufficient to impose the complete range of the dI2 cell fate.

The expression of Lhx2 following Lhx9 ectopic expression was used to study dI1 cell fate acquisition. Neurons expressing Lhx9 down-regulate the expression of Lhx2 (Figure 7C, D), suggesting that the segregation to dI1_ipsi _neurons, expressing Lhx9, and dI1_comm _neurons, expressing Lhx2_high_/Lhx9_low_, is mediated by cross-repression between Lhx9 and Lhx2. This hypothesis is supported by the reciprocal downregulation of Lhx9 via ectopic expression of Lhx2 (YH and AS, data not shown). Atoh1 is expressed in dI1 progenitor neurons, and its expression is down-regulated in differentiated post-mitotic dI1 neurons. Ectopic expression of Lhx9 causes down-regulation of Atoh1 (Figure 7E, F), suggesting that Lhx9, when up-regulated in post-mitotic dI1 neurons, is a repressor of Atoh1. *Barhl1*/*2 *genes are expressed in rodent dI1 neurons [31]. However, the orthologous genes are not present in the chick genome. Due to the lack of post-mitotic dI1 markers, the possible repression of dI2 cell fate was studied following Lhx9 ectopic expression. Foxd3 is not repressed in Lhx9-expressing neurons (Figure 7G, H). Lhx9 is thus insufficient for repressing dI2 cell fate. Thus, Lhx9 and Lhx1 cross-repress each other without changing the complete range of cell fate identity. Lhx9 and Lhx1 may nevertheless control some features of differentiated dI neurons. The role of Lhx9 and Lhx1 in axon guidance was studied in the following experiments.

### Changing the Lim-HD code of dI1 and dI2 neurons by ectopic expression – general considerations

The repression of endogenous Lim-HD following ectopic expression of a reciprocal Lim-HD gene results in replacement of the Lim-HD code. To study the role of the Lim-HD code in the assignment of the axonal projection pattern of dI1 and dI2 neurons, their Lim-HD code was alternated. The following considerations were taken into account in the subsequent ectopic expression experiments. First, to study cell autonomous effects, Lim-HD proteins were expressed specifically in the reciprocal dI neurons utilizing EdI enhancers (Lhx9 in dI2, and Lhx1 in dI1). Second, to follow the axonal trajectories of the manipulated neurons, taumyc or GFP were co-expressed with the ectopic Lim-HD protein from the same plasmid. Third, ectopic expression of Lim-HD may lead to a change in cell properties and, subsequently, to its own down-regulation. For example, Lhx9 expressed in dI2 utilizing the EdI2/V1 enhancer may up-regulate certain dI1 characteristics, ultimately leading to down-regulation of the EdI2/V1 enhancer. The stable Cre/Lox systems were used to stabilize the ectopic expression. Fourth, ectopic expression may result in high, non-physiological levels of exogenous protein levels. The levels of ectopic Lhx9 were compared to the endogenous levels of Lhx2 and Lhx9 (in the non-electroporated side of the neural tube). Utilizing the Cre/Lox system, the exogenous and endogenous levels of Lhx9 were similar (Additional file 4).

### Lhx1 controls caudal turning

**Figure 8:**
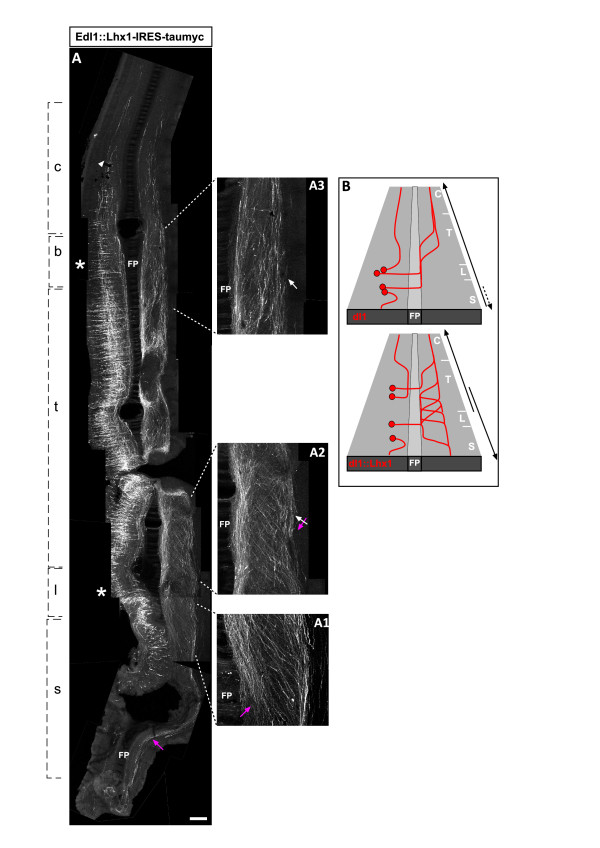
**Lhx1 confers caudal axonal projection to the caudal dI1_comm _neurons**. **(A, B) **Lhx1 and taumyc were expressed ectopically in dI1 neurons, utilizing the Cre/lox system and the EdI1 enhancer (EdI1::Cre + pCAGG-LoxP-STOP-LoxP-Lhx1-IRES-taumyc). The following dI2-specific axonal cues are assumed by the commissural dI1^Lhx1 ^neurons: at the lumbosacral levels dI1^Lhx1 ^axons turn caudally (A, A1); at the thoracic level axons turn either rostrally or caudally, forming a crisscross pattern (A, A2); at the cervical level, axons turn rostrally (A, A3). An illustration of the phenotype of dI1^Lhx1 ^ectopically expressing neurons is presented in (B). c, cervical level; b, brachial level; FP, floor plate; l, lumbar level, s, sacral level; t, thoracic level. The asterisks represent the level of the limbs. The white arrows point to the rostrally projecting axons. The magenta arrows point to the caudally projecting axons. The crossed arrows point to the crisscross axonal pattern. The arrow-head points to the longitudinal ipsi-lateral axons. Scale bar: 150 μm (A); 75 μm (A1–A3).

Lhx1 controls the projection of LMCl axons to the dorsal limb. LMC neurons that ectopically express Lhx1 settle at the lateral LMC and project their axons to the dorsal limb [4]. To test whether Lhx1 may also control the axonal projection of dI2 neurons, it was expressed ectopically in dI1 neurons. The EdI1 enhancer, driving Cre recombinase, was expressed in the neural tube along with an Lhx1/taumyc Cre-conditional plasmid (pCAGG-LoxP-STOP-LoxP-Lhx1-IRES-taumyc; Figure 8; Table 1).

The commissural dI1 and dI2 axonal patterning differ in two aspects (Figures 3, 4 and 5): in the lumbosacral neural tube all dI2_caud _axons project caudally, while only the caudal sacral dI1_ipsi _axons project caudally; and at the caudal third of the thoracic level, dI2 axons turn either rostrally or caudally, forming a 'crisscross' axonal pattern at the contra-lateral side, while dI1_comm _axons turn only rostrally. The consequence of ectopic Lhx1 expression in dI1 neurons (dI1^Lhx1^) was studied, focusing on the above features (Figure 8). At the lumbosacral level dI1^Lhx1 ^axons turn caudally (Figure 8A, A1). At the caudal thoracic level a crisscross pattern of axons turning either rostrally or caudally is evident at the contra-lateral side of the neural tube (Figure 8A, A2). Hence, all the dI2_caud _axonal features are assumed by the commissural dI1^Lhx1 ^neurons (Figure 8B).

Longitudinal axonal tracks of dI1^Lhx1 ^are present at the ipsi-lateral side (Figure 8A). Hence, Lhx1 does not suppress the ipsi-lateral projection of dI1_ipsi _neurons. However, a dI1^Lhx1 ^fascicule is observed at the ipsi-VF and the ipsi-LF, while dI1_ipsi _axons form only an ipsi-LF bundle. The ipsi-VF is a characteristic of V1 axons [29] (Figure 4A), which also express Lhx1. Thus, Lhx1 is sufficient to impose dI2-like and V1-like axonal trajectories to dI1_comm _and dI1_ipsi _neurons, respectively.

### Lhx9 controls rostral turning

**Figure 9:**
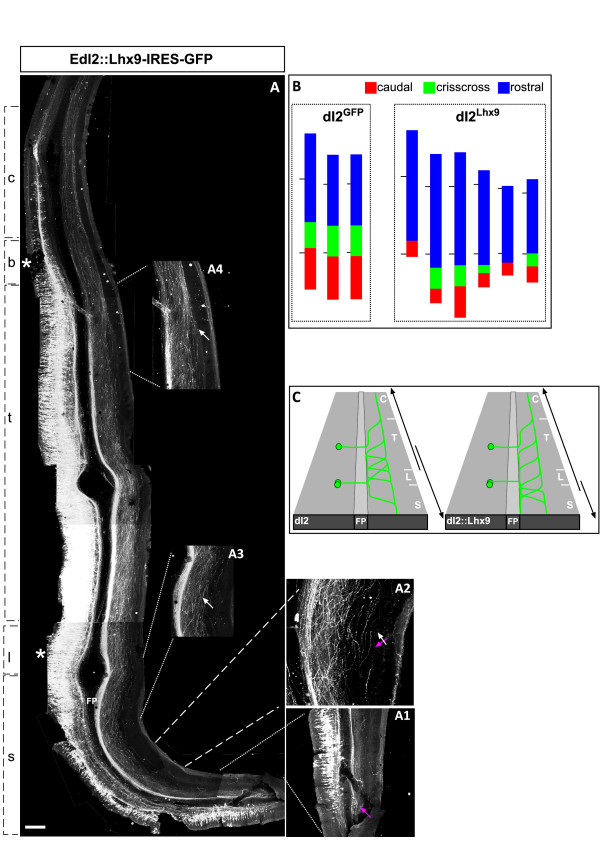
**Lhx9 mediates a caudal to rostral change in the turning of dI2 axons**. Lhx9 and GFP were expressed ectopically in dI2 and V1 neurons, utilizing the Cre/lox system and the EdI2/V1 enhancer (EdI2/V1::Cre + pCAGG-LoxP-STOP-LoxP-Lhx9-IRES-GFP). **(A) **At the caudal sacral level the commissural dI2^Lhx9 ^axons turn caudally (A, A1). At the rostral sacral level axons turn rostrally and caudally, forming a crisscross pattern (A, A2). Rostral to the lumbar level dI2^Lhx9 ^axons turn rostrally (A, A3, A4). A longitudinal fascicle at the ventral funiculus (VF) is present at the ipsi- and contra-lateral sides of the floor plate (A, A4). **(B) **Schematic representation of the caudal/rostral axonal projection of three control neural tubes (dI2^GFP^) and of six manipulated neural tubes (dI2^Lhx9^). The vertical lines represent the location of the limbs. **(C) **Schematic illustration of the phenotype of dI2^Lhx9 ^axonal cues. c, cervical level; b, brachial level; FP, floor plate; l, lumbar level, s, sacral level; t, thoracic level. The asterisks represent the level of the limbs. The white arrows point to the rostrally projecting axons. The magenta arrows point to the caudally projecting axons. The crossed arrows point to the crisscross axonal pattern. Scale bars: 200 μm (A); 150 μm (A1, A3–A5); 100 μm (A2).

Lhx9 was expressed in dI2 and V1 neurons utilizing the Cre/LoxP systems (EdI2/V1::Cre + pCAGG-LoxP-STOP-LoxP-Lhx9-IRES-GFP; Figure 9; Table 1). The dI2-specific axonal trajectories at the contra-lateral side are described below. Lhx9/GFP-expressing dI2 neurons (dI2^Lhx9^) project their axons both rostrally and caudally, forming caudally projecting fascicules at the caudal neural tube level (Figure 9A, A1), followed by a crisscross pattern and rostrally projecting axons (Figure 9A, A2). However, the transition point between caudal, crisscross and rostral projection has been shifted caudally. Wild-type dI2 axons turn caudally from the lumbar level, while dI2^Lhx9 ^axons turn caudally only in the caudal-sacral level (Figure 9A, A2). The crisscross pattern of dI2 axons is limited to the caudal third of the thoracic level, while dI2^Lhx9 ^axons either form a crisscross pattern at the rostral-sacral level (four out of six embryos), or do not form it at all (two out of six embryos) (Figure 9B). The rostral turning, which is restricted to levels that lie rostrally to the caudal third of the thoracic level, is expended caudally to the entire thoracic level (five out of six embryos), and even to the lumbar level (three out of six embryos) (Figure 9B). Thus, Lhx9 appears to activate rostral turning at the expense of caudal turning (Figure 9C). A similar axonal patterning was observed when Lhx2 (four embryos; Table 1; Additional file 5) or Lhx2 plus Lhx9 were expressed in dI2 neurons (one embryo; Table 1; Additional file 6). Hence, dI2^Lhx9 ^axonal trajectories are a mixture of dI1 and dI2 axonal cues. While there are fewer caudally projecting dI2^Lhx9 ^axons than dI2 axons, caudally projecting axons remain more prevalent than dI1 axons.

### Lhx9 controls the dorsoventral position at which axons turn into the longitudinal plane

**Figure 10:**
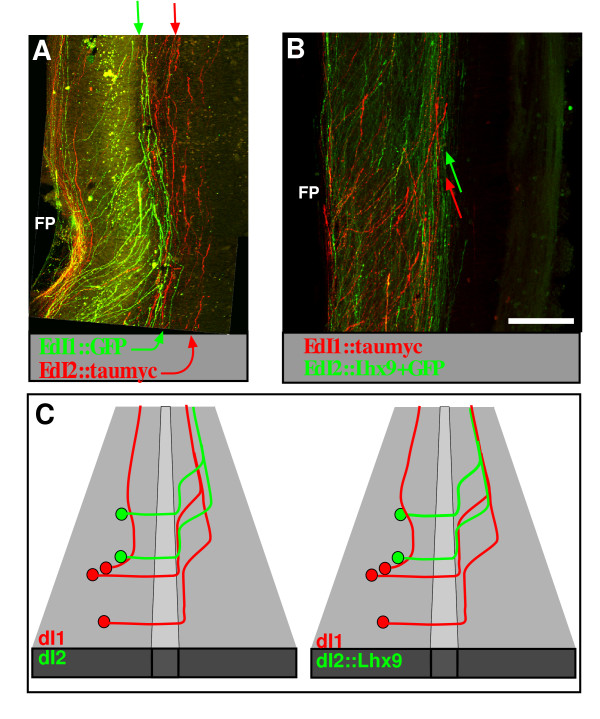
**Lhx9 triggers a ventral shift to the longitudinal dI2^Lhx9 ^axons**. **(A, B) **An open-book preparation of the neural tube in which the commissural dI1 axons express GFP and dI2 axons express taumyc (EdI1::Gal4 + UAS::GFP; EdI2/V1::Cre + pCAGG-LoxP-STOP-LoxP-taumyc). The contra-lateral side of the neural tube is shown. dI2 axons turn longitudinally, at the lateral funiculus (LF), and form a fascicle that is located dorsally to the dI1 fascicle. Ectopic expression of Lhx9 in dI2 neurons (EdI2/V1::Gal4 + UAS::GFP_UAS::Lhx9) resulted in dI2^Lhx9 ^axons fasciculating with dI1 axons at the contra-LF. **(C) **Schematic illustrations of the co-fasciculation phenotype of dI2^Lhx9 ^neurons. The green and red arrows point to the axons of dI1 and dI2 neurons according to the color code that is indicated in each illustration. Scale bars: 150 μm (A, C, D); 200 μm (B).

Next, we focused on the possible role of Lhx9 on the topographic organization of the longitudinal axonal tracks at the LF. The homophilic fasciculation of the dI1_ipsi _+ dI1_comm _axons at the LF may imply that the longitudinally projecting bundle of dI1 and dI2 axons forms a distinct and specific fascicule. To map the topographic organization of dI1 and dI2 longitudinal tracks, dI1 and dI2 neurons were labeled unilaterally, using the Cre/LoxP method for the commissural dI2 neurons, and the Gal4/UAS method for the dI1 neurons (Figure 10A). Expression of reporter genes in dI1 and dI2 axons reveals that the longitudinal dI2 fascicule at the LF is dorsal to the dI1 fascicle (Figure 10A, C).

The relative position at the LF of dI2^Lhx9 ^axons was compared to dI1 axons. Taumyc was expressed under the control of the EdI1 enhancer (EdI1::Cre + pCAGG-LoxP-STOP-LoxP-taumyc), together with ectopic Lhx9 in dI2 neurons (EdI2/V1::Gal4 + UAS::Lhx9_UAS::GFP). dI2^Lhx9 ^axons turn rostrally at the LF, together with dI1_comm _axons (Figure 10B, C). The specific bundle of dI2 axons at the LF was not formed, and dI2 and dI1_comm _axons intermingled and formed one fascicle as they turned longitudinally at the LF (Figure 10E, Table 1). Thus, Lhx9 may control the homophilic interaction with dI1 axons along their longitudinal projection toward the brain. Alternatively, Lim-HD code may control the position of the LF along the dorsal/ventral axis, where Lhx9 directs a more ventral position than Lhx1, and the mis-expression of Lhx9 in dI2 axons shifts the dorsoventral position at which dI2 axons turn into the longitudinal plane.

## Discussion

Cell fate acquisition is manifested by the activation of transcription factors. Many features define the function of a neuron, including cell body positioning, dendritic tree morphology, axonal projection, neurotransmitters specificity and excitatory or inhibitory output. The specification of neurons might be governed by linear sequential activation of transcription factors, or by activation of a parallel pathway, each one driving a specific neuronal characteristic. In the current study we have combined molecular and morphological tools to follow the development and axonal patterning of molecularly defined groups of dorsal spinal interneurons. We provide evidence that the Lim-HD proteins Lhx1 and Lhx9 are sufficient to influence axonal patterning without affecting neuronal fate.

### Diversity of dI axonal projections

The combination of specific enhancers, augmentation of expression levels utilizing the Cre/LoxP and the Gal4/UAS systems, and chick electroporation provide quick and efficient tools for deciphering axonal pathways of a genetically defined group of neurons. The emerging picture is of a complex divergence of axonal cues that arises from dI1 and dI2 subpopulations. dI1 and dI2 give rise to two subpopulations each, defined by the direction of their axonal projections. The simultaneous molecular and spatially restricted labelling of two neuronal populations, dI1 + dI2 and dI1_ipsi_+ dI1_comm_, underscores the axonal architecture of dI1 and dI2 axons: dI1_ipsi _and dI1_comm _fasciculate together at the LF; dI1 and dI2 axonal tracks at the LF are segregated.

The axonal pathways of spinal internerons were mapped previously utilizing diI injection [32-34]. Kadison and Kaprielian described four main axonal projections of decussating axons: intermediate longitudinal commissural (ILC), medial longitudinal commissural (MLC), bifurcating longitudinal commissural (BLC), and forked transverse commissural (FTC) [34]. ILC axons travelled rostrally in an arcuate manner, extending into VF regions of the spinal cord before executing a second turn into the longitudinal plane at the LF. The contra-laterally projecting dI1 and dI2 neurons project their axons in an ILC pattern. MLC axons, which extend along the floor plate boundary at the VF for distances greater than 100 mm, BLC axons, which bifurcate to rostral and caudal projections, and FTC axons, which form a trident-shaped or forked projection, were not identified in the current study. The labelling of multiple neurons achieved using the electroporation paradigm in the current study may obscure these projection patterns. A moderate number (10%) of decussated axons was observed to extend in the caudal direction following DiI injection [34]. However, our studies point to a larger quantity of caudally projecting neurons. At the sacral level dI1_ipsi _and all dI2_caud _axons extend caudally. At the lumbar region and the caudal third of thoracic levels, about half of dI2 axons project caudally. Hence, a rostral to caudal stepwise increase in caudal projection is evident. Injection of DiI into neurons at the sacral level may reveal more caudally projecting axons.

### Transcriptional control of axonal guidance

The divergence in axonal growth along the ipsi/contra and the caudal/rostral axes may stem from a cell type-specific expression of transcription factors. Namely, dI1_comm _and dI2_comm _genes could theoretically be expressed in the contra-laterally projecting dI1_comm _and dI2 neurons, respectively. A possible candidate for such a mechanism is Lhx2, which is expressed only in dI1_comm _neurons. However, gene-targeting experiments of Lhx2 and Lhx9 have shown that a Lim-HD code does not control ipsi- versus contra-lateral axonal projection [16]. Similarly, Lhx1 is expressed in the ipsi-only population V1 and the contra-mostly population dI2. Therefore, Lhx1 is probably not implicated in controlling of the contra-lateral projection of dI2 neurons. Alternatively, common *dIcomm *and *dIipsi *genes might be expressed in all the dIN_comm _and dIN_ipsi _neurons, respectively. Transcription factors such as Unc4 and NSCL1, which are expressed in all interneurons [35,36] in an overlapping pattern to the commissural-only genes *TAG1 *and *Robo3*, are candidates for controlling commissural guidance choice of dIs.

A similar transcriptional mechanism may account for the caudal versus rostral axonal choice. A transcriptional code may discriminate between the longitudinal levels. Hence, the combination of *dIcaudal*, expressed at the caudal neural tube, and *Lhx1 *may confer caudal projection. Potential *dIcaudal *and *dIrostral *factors may be the Hox proteins. A Hox code determines the rostral/caudal identity of motor neurons, and the combination of Hox and Lim-HD codes determines the subclassification of motor neuron pools [37,38]. The caudally expressing *Hox10 *and *Hox11 *genes may confer caudal turning to the lumbosacral dI2 neurons, while the more rostral Hox, *Hox8 *and beyond, may confer rostral turning. The caudal thoracic crisscross pattern might be controlled by *Hox9+*/*Hox8*- code. The rostral to caudal change in axonal projection, following Lhx1 ectopic expression, may place Lhx1 as a positive activator of the *Hoxcaudal *genes *Hox10 *and *Hox11*. Complementarily, Lhx9 may suppress caudal turning by suppressing *Lhx1 *and/or *Hox10 *and *Hox11 *expression. Alternatively, Lhx9 may suppress *Hox10 *and *Hox11 *expression, and Lhx1 may reveal Hox10 and Hox11 activity by suppressing their suppressor, Lhx9.

What are the axonal cues that may govern caudal turning? Axons may turn in different directions due to different axonal cues or differential responsiveness to common cues. The differential cues theory is not supported by our data. At the caudal thoracic levels dI2 axons, at the same rostro/caudal level, turn either rostrally or caudally. The conversion in axonal directionality may be governed cell autonomously by receptors or signalling molecules that convert attraction to repulsion. The rostral turning of commissural neurons along the floor plate is mediated by increasing caudal-to-rostral levels of Wnt proteins [39], which attract axons; and decreasing caudal-to-rostral levels of Shh [40], which repel axons. Caudally turning neurons may express receptors or signalling molecules that convert Wnt attraction to repulsion and/or Shh repulsion to attraction. *In vitro *assays with caudal dI2 neurons challenged with Wnts and Shh should clarify whether a cell autonomous change in responsiveness governs dorsal and caudal turning.

### Role of Lim-HD in cell fate determination

The emergence of interneuron divisions is marked by a mutual exclusion in the expression profile of bHLH proteins and Lim-HD proteins. Progenitor dI1/2 neurons express Atoh1 and Ngn1/2, respectively [13]. Loss and gain of function experiments have demonstrated that these proteins cross repress each other, and are both required and sufficient for the differentiation of dI1/2 neurons [18,41]. Therefore, in the absence of Atoh1, dI1 neurons fail to differentiate, and are converted to dI2 neurons [18]. Lim-HD genes, expressed in the post-mitotic dI1/2 neurons, are probably activated by the bHLH proteins. Our ectopic expression experiments demonstrate that Lim-HD proteins also cross-repress each other in dI1 and dI2 neurons. Thus, the distinct identity of adjacent neurons is guaranteed at the mitotic and post-mitotic stages by cross-repression of bHLH and Lim-HD proteins, respectively. Loss of function experiments have demonstrated that in the absence of Lhx2/9 or Lhx1/5, the fate of dI1 and dI2 neurons is not altered [16,42]. In the Lhx2/9 double knockout mouse, dI1 cells express dI1-specific genes, and the Lim-HD code is not changed to Lhx1/5. It is conceivable that Atoh1, which acts upstream to Lhx2/9, is repressing Ngn1/2 and thus indirectly prevents the activation of Lhx1/5. It is also possible that bHLH proteins control dI1/2 cell fate by activating Lim-HD proteins and, in addition, in a feed forward mechanism, directly control cell fate. Thus, the elimination of Lim-HD can be compensated for by bHLH proteins. Ectopic Lim-HD proteins may play a dominant role in repressing other Lim-HD proteins and in repressing bHLH protein activity. This assumption is supported by the observation that ectopically expressed Lhx1 suppresses the expression of Atoh1 (YH and OA, unpublished results). The Lim-HD proteins Isl1 and Lhx1 play a similar role in determining the fate of LMC neurons. Retinoic acid induces LMCl neurons by activating Lhx1 and repressing Isl1 expression. In the absence of Lhx1, LMCl neurons differentiate, settle at the lateral LMC column and do not upregulate Isl1 expression. Thus, like bHLH proteins in dI1/2 neurons, retinoic acid is sufficient to confer LMCl identity, probably by bypassing Lhx1 signalling in a feed-forward mechanism [4,5].

## Conclusion

The emergence of interneuron divisions is marked by mutual exclusion in the expression profile of bHLH and Lim-HD proteins [13]. Loss of function experiments have demonstrated that in the absence of Lhx2/9 or Lhx1/5, the fate of dI1 and dI2 neurons is not altered [16,42]. We have used targeted ectopic expression to explore the role of the Lim-HD proteins Lhx9 and Lhx1 in patterning the axonal trajectories of dI1 and dl2 neurons. Our results point to a new role of Lim-HD proteins in controlling the longitudinal turning choice and axonal sorting of dI1 and dI2 neurons.

## Materials and methods

### *In ovo *electroporations

Fertilized white Leghorn chicken eggs were incubated at 38.5 to 39°C. A DNA solution of 5 mg/ml was injected into the lumen of the neural tube at either HH stage 12 to 14 (cytomegalovirus (CMV) enhancer in pCAGG plasmid) or stage 17 to 18 (EdI1 and EdI2/V1 enhancers). For double-sided electroporation, a 1-h interval interceded between the first and second electroporations.

Electroporation was performed using three 50 ms pulses at 25V, applied across the embryo using a 0.5 mm Tungsten wire and a BTX electroporator (ECM 830). Embryos were incubated for 2 to 3 days prior to analysis.

### Strategies for cell type specific expression

#### Testing enhancer specificity

Co-expression of a plasmid containing an enhancer driving the expression of Cre recombinase and a reporter plasmid in which a floxed *mCherry *gene was inserted between the CAGG enhancer/promoter module and the *GFP *gene (pCAGG-LoxP-mCherry-LoxP-GFP) was performed. Cells that do not express Cre (general expression) will express mCherry, while cells that express Cre under the control of the specific enhancer will express GFP. The CAGG enhancer is not restricted either spatially or temporally, while expression from the specific enhancer is initiated in post-mitotic cells. Thus, residual expression of mCherry is observed in the GFP-positive cells.

#### Testing cell type specificity of an enhancer

Co-expression of a plasmid containing an enhancer driving the expression of Cre recombinase and a reporter plasmid in which a transcriptional STOP module was inserted between the CAGG enhancer/promoter module and the *nuclearGFP *gene (pCAGG-LoxP-STOP-LoxP-nGFP) was performed. Embryos were electroporated at stage 16 since earlier electroporation may yield non-specific expression [22]. Embryos were analyzed at stage 23 to 24.

#### Mapping axonal trajectories using the Cre/Lox system

Conditional GFP (pCAGG-LoxP-STOP-LoxP-GFP) or taumyc (pCAGG-LoxP-STOP-LoxP-taumyc) plasmids were electroprated along with enhancer::Cre plasmid. The entire spinal cord was excised at E6 and was prepared as an open-book for further analyses.

#### Mapping axonal trajectories using the Gal4/UAS system

The Gal4 DNA binding domain fused to an activation domain [43] was cloned downstream of the dI specific enhancers. The enhancer::Gal4 plasmid was co-electroporated with a UAS::GFP plasmid.

#### Enhancer intersection technique

The expression of the reporter gene *GFP *is dependent on both Gal4 and Cre. A floxed STOP cassette was inserted between UAS and GFP (UAS-LoxP-STOP-LoxP-GFP). Hence, removal of the STOP cassette by Cre recombinase, and activation of transcription by Gal4 are required for GFP expression. The intersection between two expression patterns is attained by electroporation of three plasmids: Enhancer1::Cre, Enhancer2::Gal4, and UAS-LoxP-STOP-LoxP-GFP.

### Spinal cord open-book preparation

E6 electroporated chick spinal cord tissues were prepared as an open-book preparation by making a longitudinal incision along the roof plate with a sharp tungsten microneedle from the hindbrain down to the tail. The dorsal root ganglia (DRGs) were then separated from the spinal cord, leaving the floor plate intact. The hind and forelimb were marked with charcoal powder, and then the spinal cord was detached from the body and fixed in 4% paraformaldehyde in phosphate-buffered saline for 1 h at room temperature, after which the tissue was spread open to produce flat-mount preparations.

### Immunohistochemistry

Embryos were fixed overnight at 4°C in 4% paraformaldehyde/0.1 M phosphate buffer, washed twice with phosphate-buffered saline, incubated in 30% sucrose/phosphate-buffered saline for 24 h, and embedded in Optimal Cutting Temperature solution (OCT). Cryostat sections (14 μm) were collected on Superfrost Plus slides and kept at -70°C. Antigen retrieval was used for paraffin sections. Sections were treated with boiled 10 mM citric acid, pH 6, for 10 minutes in the microwave. The following antibodies were used: rabbit polyclonal GFP antibody (Molecular Probes, Eugene, Oregon, USA)), Pax2 (Abcam, Cambridge, MA, USA)), myc (9E10), Isl1 (4D5), Lhx1/5 (4F2), Engrailed, Lhx2/9 (rabbit serum) (all provided by T Jessell, Columbia University, New York, NY, USA). Cy2, RRX and cy5 were used as fluorochromes. Images were taken under a microscope (Axioscope 2; Zeiss) with a digital camera (DP70; Olympus) or confocal microscope (FV1000; Olympus).

### DNA

The EdI1 enhancer element was amplified by PCR from a genomic mouse DNA utilizing the primers [ATGAGCTCATCCCTTTTTGCTCCCTCAC] and [ATGCTAGCGGTGTTGTGGTTGACAGCAG]. EdI2/V1 was amplified utilizing the primers [ATGAGCTCGCTCTCTCTGCCTACCTCAGC] and [ATGCTAGCAACCTAGTGCCCTTGCACAC]. The enhancers were cloned into 5'*Sac*I/3'*Nhe*I sites of the appropriate Cre and Gal4 plasmids [23]. The 13G Ngn1 enhancer (EdI1/2) was generated by PCR from a genomic mouse DNA utilizing the primers [ATTGCGGCCGCATCAGGCGCCGGATCACTTTG] and [GATCTAGACCTTCACCATCGTTAACACTGG] and cloned into 5'*Not*I/3'*Xba*I sites of the Cre plasmid. Chick Lhx2 and Lhx9 were obtained from Tom Jessell. Chick Lhx1 was obtained from Artur Kania. Chick Foxd3 and Atoh1 were obtained from the chick expressed sequence tag sequencing project [44].

### *In situ *hybridization

Antisense digoxigenin-labeled probes were prepared by *in vitro *transcription (Roche, Nutley, NJ, USA)). *In situ *hybridization was performed on electroporated sections and was combined with immunohistochemistry. Prior to the *in situ *hybridization, sections were incubated with primary GFP antibody, and then a standard *in situ *protocol was applied, followed by a secondary fluorescent antibody treatment. Alternatively, adjacent sections were collected on different slides. One set of slides was used for *in situ *hybridization, and the other for immuno-detection of GFP.

## Abbreviations

bHLH: basic helix loop helix; BLC: bifurcating longitudinal commissural; E: embryonic day; FTC: forked transverse commissural; GFP: green fluorescent protein; ILC: intermediate longitudinal commissural; LF: lateral funiculus; MLC: medial longitudinal commissural; nGFP: nuclear GFP; VF: ventral funiculus.

## Competing interests

The authors declare that they have no competing interests.

## Authors' contributions

OA performed the experiments presented in Figures 1, 2, 3, 4, 5, 6 and 8, 9, 10 and in Additional files 1, 2, 3, 5 and 6. YH preformed the experiments presented in Figures 6 and 7 and in Additional file 4. LV helped in the experiments presented in Additional file 1. AS preformed the experiments presented in Figure 7. SZ helped in the experiments presented in Figure 9. AV characterized in mouse the enhancers that were used in Figure 1. All authors participated in the preparation of the manuscript.

## Supplementary Material

Additional file 1 **EdI1/2 enhancer drives expression in dI1 and dI2 neurons**. The EdI1/2 enhancer element was cloned upstream of Cre recombinase and electroporated with a conditional nuclear GFP (CAGG-loxP-STOP-loxP-nGFP) plasmid. Chick embryos were electroporated at stage 16 and fixed at stage 23. Cross-sections of electroporated neural tube were stained with dI-specific antibodies. Neurons expressing nGFP are Lhx1/5^+^/Pax2^- ^(A) and Lhx2/9^+^/Isl1^-^. The boxed areas are represented as in their different channels at the right side of each panel. The arrows point to the dI2 neurons that express nGFP, and the arrowheads to dI1 neurons expressing nGFP. Scale bars: 50 μm. Click here for file

Additional file 2 **Rostral versus caudal turning of dI2 axons**. The contra-lateral side of **(A-D) **cervical and **(E-H) **sacral levels of four different embryos expressing GFP in dI2 neurons (A, B, E, F) or dI2/V1 neurons (C, D, G, H). The vast majority of the axons at the cervical level are dI2_rost_, and at the sacral level dI2_caud_. FP, floor plate. Scale bar: 150 μm (A, B, F); 200 μm (C, E, G, H); 250 μm (D). Click here for file

Additional file 3 **Most dI2 neurons are commissural**. **(A-C) **Cervical and **(D) **brachial levels of four different neural tubes obtained from chick embryos were electroporated at stage 16 with three plasmids: dI1/2::Cre, dI2/V1::Gal4 and UAS::LoxP-STOP-LoxP-GFP. Only dI2 neurons expressed GFP. Only in one embryo (A) are ipsi-lateral longitudinal axons that project rostrally visible. For quantification, EGFP brightness intensity at the ipsi- and contra-lateral sides was measured utilizing NIH image software. Scale bar: 150 μm (A, C); 200 μm (B, D). Click here for file

Additional file 4 **The level of ectopic Lhx9 protein is similar to the levels of the endogenous protein**. Lhx9 was expressed **(A) **uniformly (pCAGG-Lhx9-IRES-nGFP), or **(B) **in dI2 neurons (EdI2/V1::Cre + pCAGG-LoxP-STOP-LoxP-Lhx9-IRES-GFP). Sections were stained with Lhx2/9 antibody. The levels of the exogenous Lhx9 are similar to the endogenous levels. Arrows point to cells with high level expression, and arrowheads to cells with low level expression. White indicates endogenousand yellow exogenous Lhx9 proteins. Scale bar: 100 μm. Click here for file

Additional file 5 **Lhx2 mediates a caudal-to-rostral change in the turning of dI2 axons**. Lhx2 + taumyc were expressed ectopically in dI2 neurons, utilizing the Cre/lox system and the EdI2/V1 enhancer (EdI2/V1::Cre + pCAGG-LoxP-STOP-LoxP-Lhx2-IRES-taumyc). **(A) **At the caudal sacral level dI2^Lhx2 ^axons turn caudally. At the rostral sacral level axons turn rostrally and caudally, forming a crisscross pattern (A, A1) (white arrows point to rostrally projecting axons and magenta arrows to caudally projecting axons). Rostral to the lumbar level dI2^Lhx2 ^axons turn rostrally (A, A2). **(B) **A schematic illustration of the phenotype of dI2^Lhx2/9 ^axonal cues. c, cervical level; b, brachial level; FP, floor plate; l, lumbar level, s, sacral level; t, thoracic level. Scale bars: 150 μm (A); 100 μm (A1, A2). Click here for file

Additional file 6 **Lhx2 + Lhx9 mediate a caudal-to-rostral change in the turning of dI2 axons**. Lhx9 + GFP and Lhx2 + taumyc were expressed ectopically in dI2 neurons, utilizing the Cre/lox system and the EdI2/V1 enhancer (EdI2/V1::Cre + pCAGG-LoxP-STOP-LoxP-Lhx9-IRES-taumyc + pCAGG-LoxP-STOP-LoxP-Lhx2-IRES-taumyc). **(A) **At the caudal sacral level dI2^Lhx2/9 ^axons turn caudally (A, A1). At the rostral sacral level axons turn rostrally and caudally, forming a crisscross pattern (A, A2). Rostral to the lumbar level dI2^Lhx2/9 ^axons turn rostrally (A, A3). **(B) **The electroporated axons co-express GFP and taumyc. **(C) **A schematic illustration of the phenotype of dI2^Lhx2/9^axonal cues. c, cervical level; b, brachial level; FP, floor plate; l, lumbar level, s, sacral level; t, thoracic level. Scale bars: 150 μm (A, B); 75 μm (A1–A3). Click here for file
