# Supplementary figures and images for: Transcriptional control of axonal guidance and sorting in dorsal interneurons by the Lim-HD proteins Lhx9 and Lhx1
Source: Neural Dev. 2009 Jun 19;4:21. doi: 10.1186/1749-8104-4-21 (PMC2704203; doi:10.1186/1749-8104-4-21)

Additional file 1

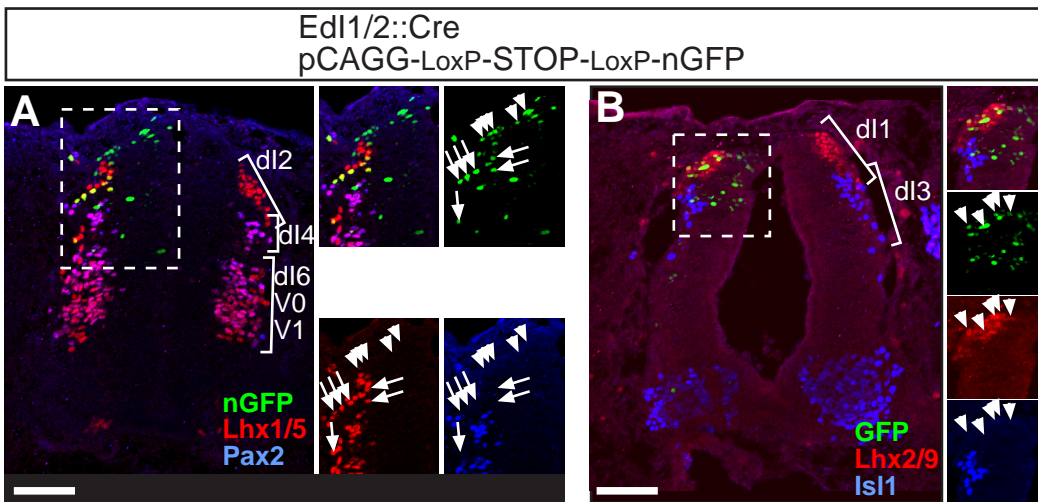

Supplement: Additional file 1 — EdI1/2 enhancer drives expression in dI1 and dI2 neurons. The EdI1/2 enhancer element was cloned upstream of Cre recombinase and electroporated with a conditional nuclear GFP (CAGG-loxP-STOP-loxP-nGFP) plasmid. Chick embryos were electroporated at stage 16 and fixed at stage 23. Cross-sections of electroporated neural tube were stained with dI-specific antibodies. Neurons expressing nGFP are Lhx1/5+/Pax2- (A) and Lhx2/9+/Isl1-. The boxed areas are represented as in their different channels at the right side of each panel. The arrows point to the dI2 neurons that express nGFP, and the arrowheads to dI1 neurons expressing nGFP. Scale bars: 50 μm. [file 1749-8104-4-21-S1.pdf]

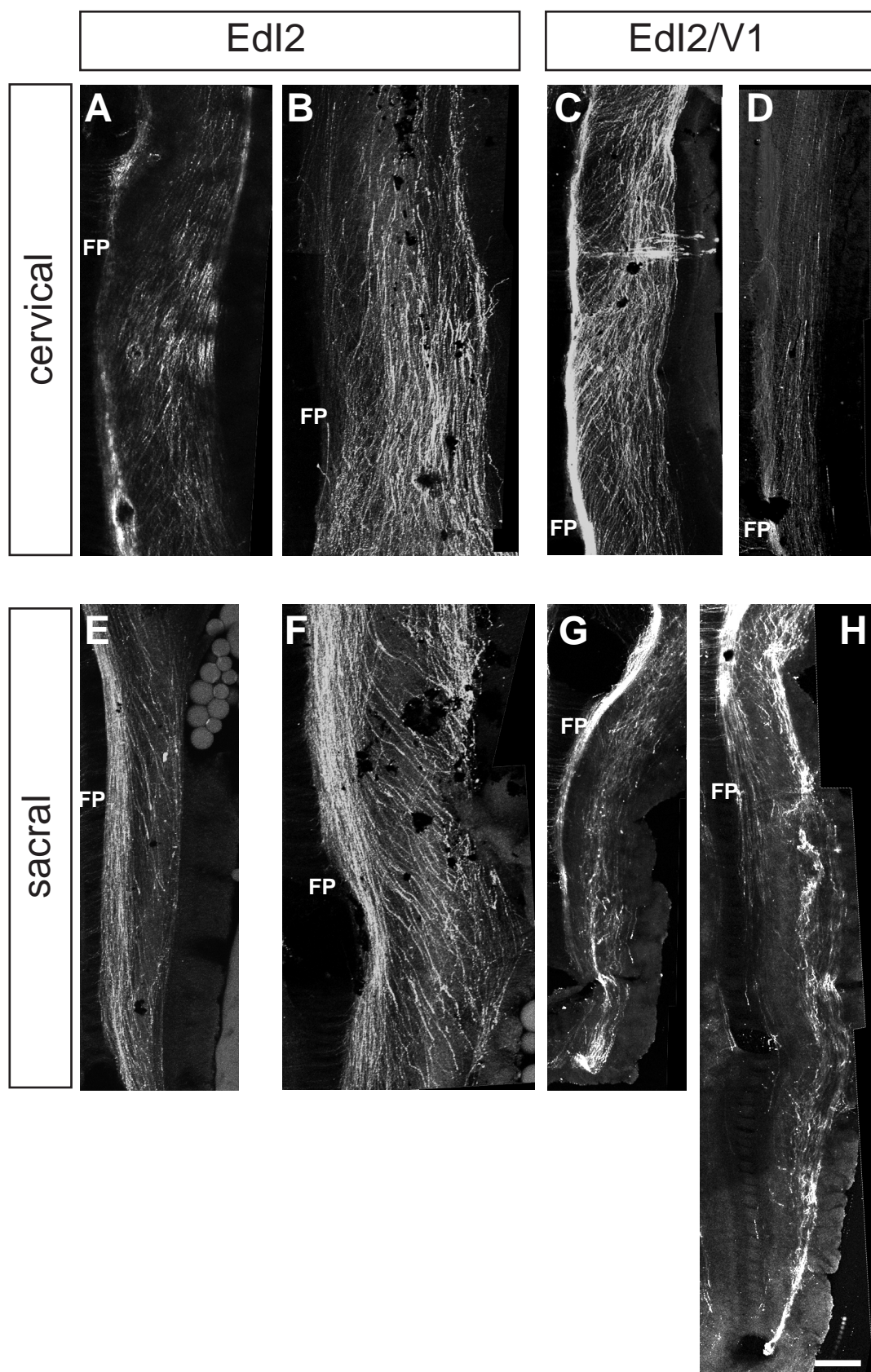

Supplement: Additional file 2 — Rostral versus caudal turning of dI2 axons. The contra-lateral side of (A-D) cervical and (E-H) sacral levels of four different embryos expressing GFP in dI2 neurons (A, B, E, F) or dI2/V1 neurons (C, D, G, H). The vast majority of the axons at the cervical level are dI2rost, and at the sacral level dI2caud. FP, floor plate. Scale bar: 150 μm (A, B, F); 200 μm (C, E, G, H); 250 μm (D). [file 1749-8104-4-21-S2.pdf]

Additional file 3

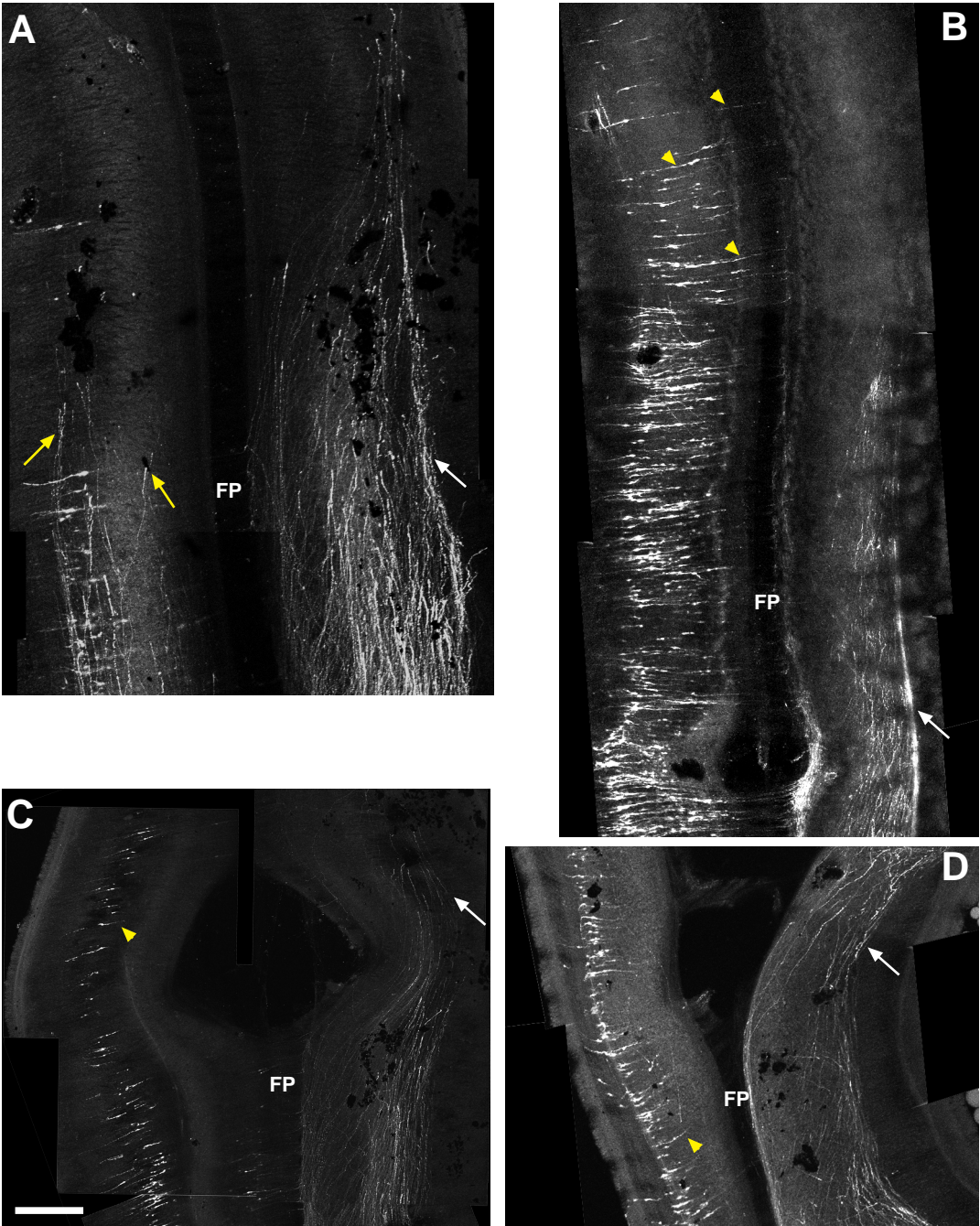

Supplement: Additional file 3 — Most dI2 neurons are commissural. (A-C) Cervical and (D) brachial levels of four different neural tubes obtained from chick embryos were electroporated at stage 16 with three plasmids: dI1/2::Cre, dI2/V1::Gal4 and UAS::LoxP-STOP-LoxP-GFP. Only dI2 neurons expressed GFP. Only in one embryo (A) are ipsi-lateral longitudinal axons that project rostrally visible. For quantification, EGFP brightness intensity at the ipsi- and contra-lateral sides was measured utilizing NIH image software. Scale bar: 150 μm (A, C); 200 μm (B, D). [file 1749-8104-4-21-S3.pdf]

Additional file 4

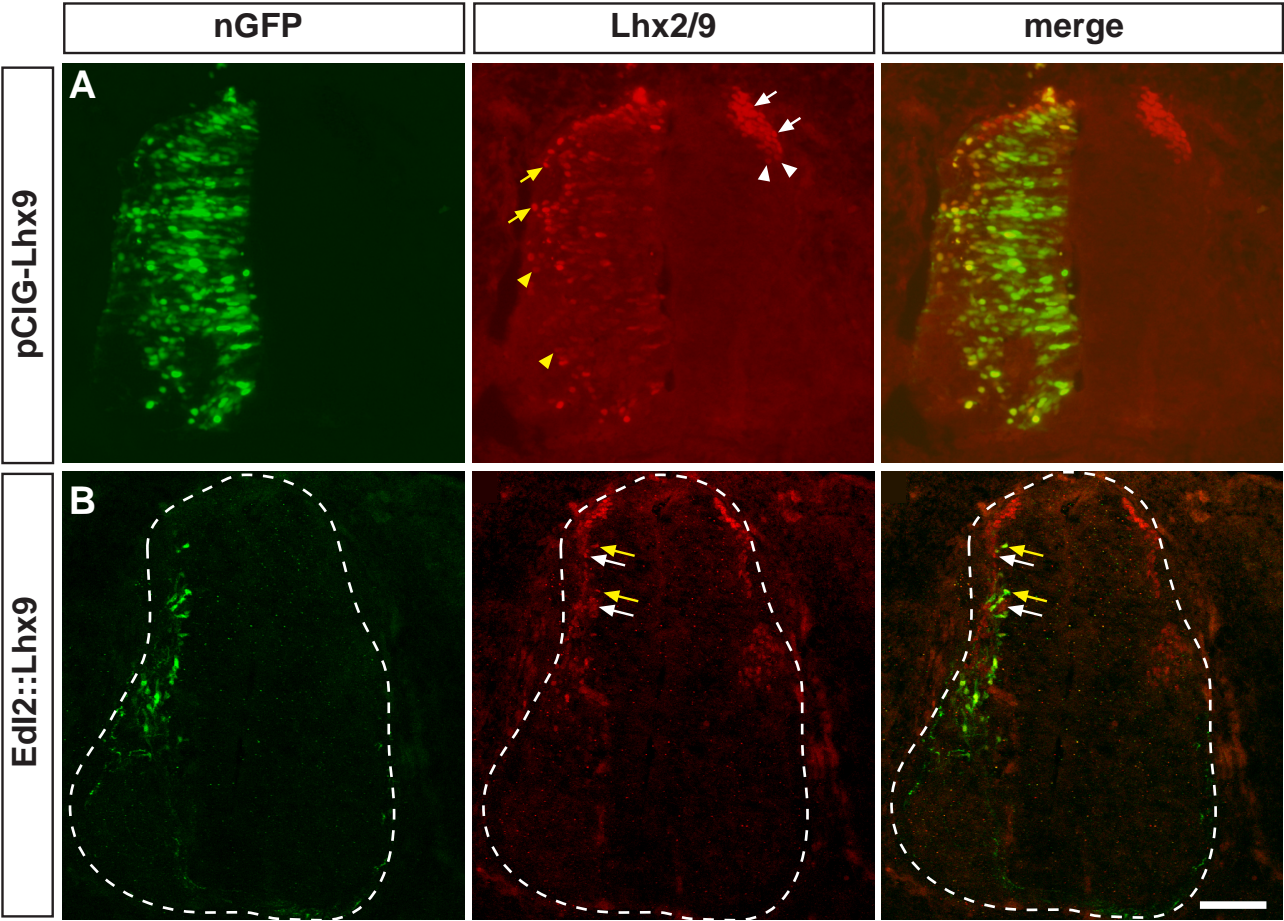

Supplement: Additional file 4 — The level of ectopic Lhx9 protein is similar to the levels of the endogenous protein. Lhx9 was expressed (A) uniformly (pCAGG-Lhx9-IRES-nGFP), or (B) in dI2 neurons (EdI2/V1::Cre + pCAGG-LoxP-STOP-LoxP-Lhx9-IRES-GFP). Sections were stained with Lhx2/9 antibody. The levels of the exogenous Lhx9 are similar to the endogenous levels. Arrows point to cells with high level expression, and arrowheads to cells with low level expression. White indicates endogenousand yellow exogenous Lhx9 proteins. Scale bar: 100 μm. [file 1749-8104-4-21-S4.pdf]

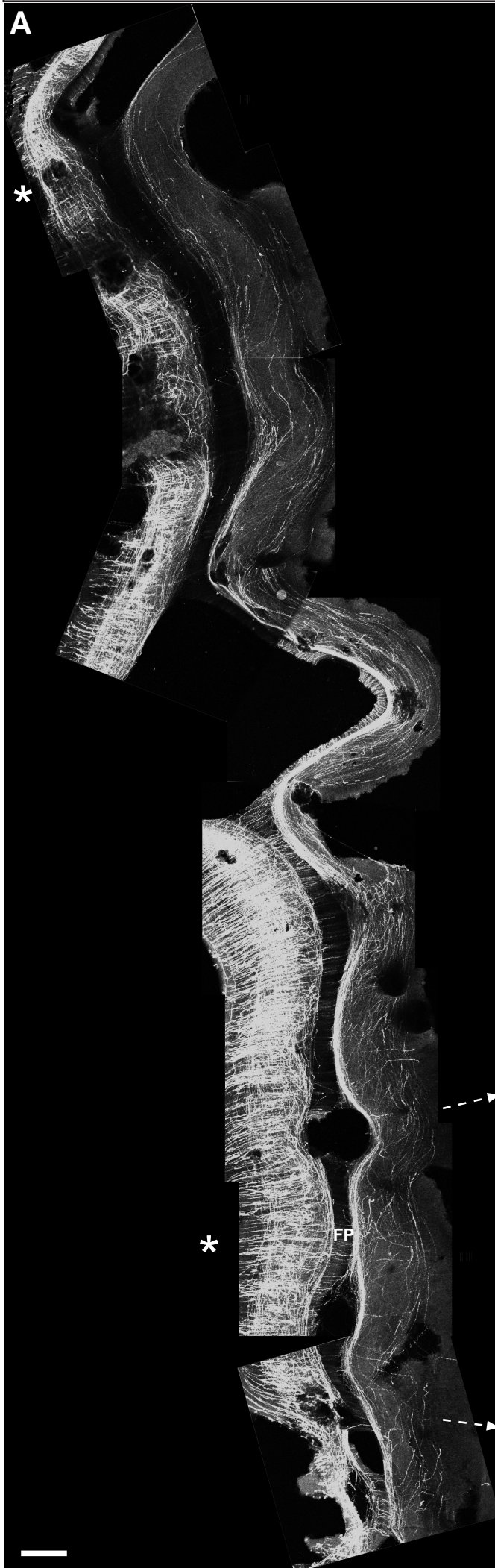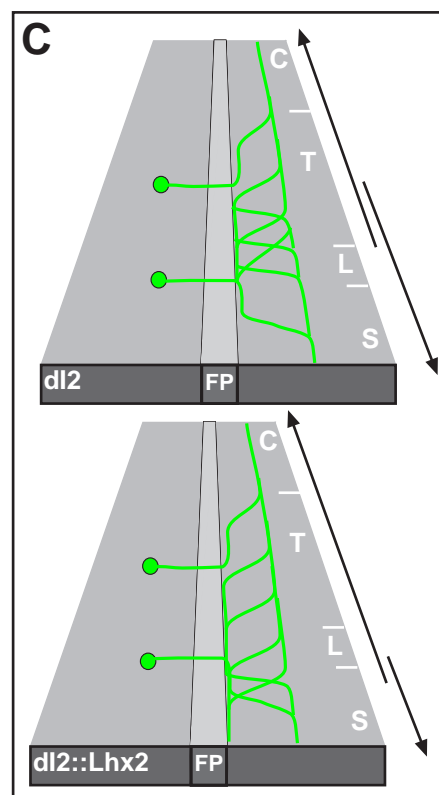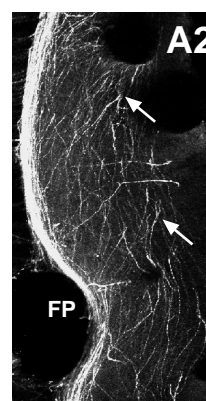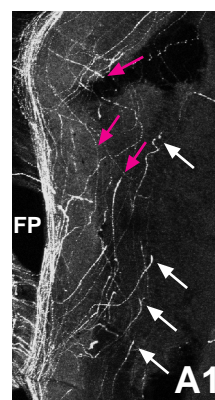

Supplement: Additional file 5 — Lhx2 mediates a caudal-to-rostral change in the turning of dI2 axons. Lhx2 + taumyc were expressed ectopically in dI2 neurons, utilizing the Cre/lox system and the EdI2/V1 enhancer (EdI2/V1::Cre + pCAGG-LoxP-STOP-LoxP-Lhx2-IRES-taumyc). (A) At the caudal sacral level dI2Lhx2 axons turn caudally. At the rostral sacral level axons turn rostrally and caudally, forming a crisscross pattern (A, A1) (white arrows point to rostrally projecting axons and magenta arrows to caudally projecting axons). Rostral to the lumbar level dI2Lhx2 axons turn rostrally (A, A2). (B) A schematic illustration of the phenotype of dI2Lhx2/9 axonal cues. c, cervical level; b, brachial level; FP, floor plate; l, lumbar level, s, sacral level; t, thoracic level. Scale bars: 150 μm (A); 100 μm (A1, A2). [file 1749-8104-4-21-S5.pdf]

Edl2::Lhx9-IRES-GFP  
Edl2::Lhx2-IRES-taumyc

Additional file 6

A

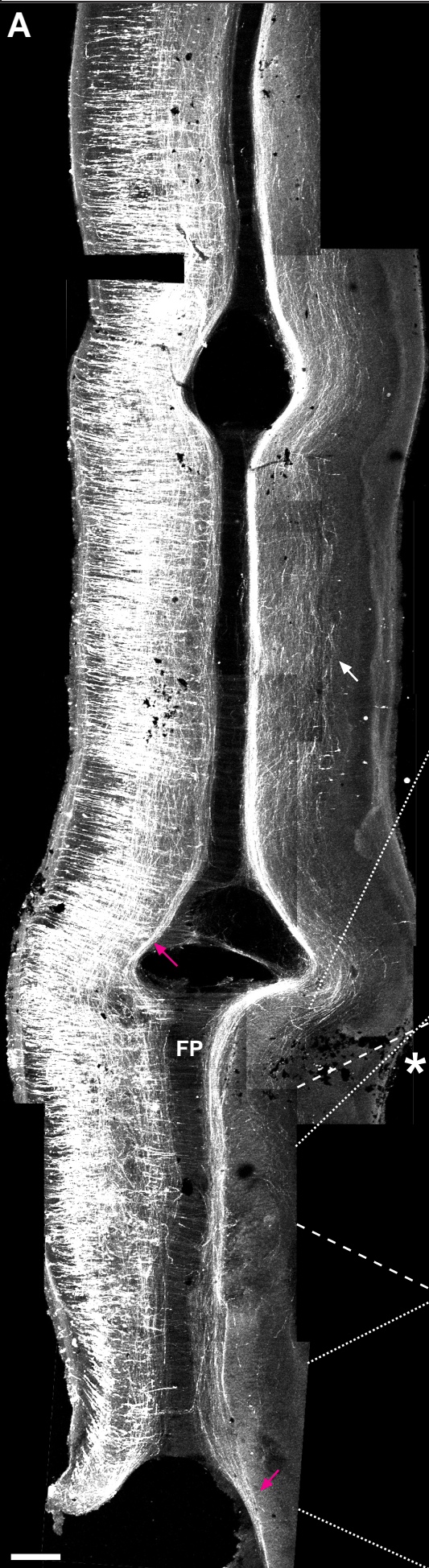

B

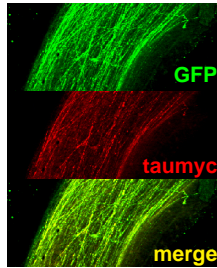

C

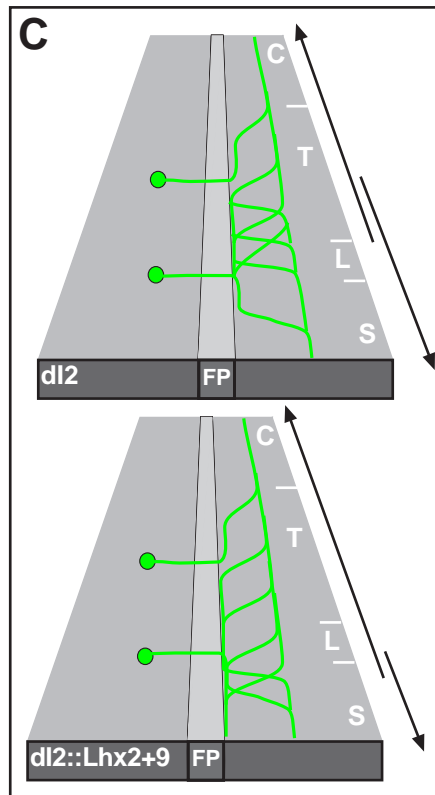

A3

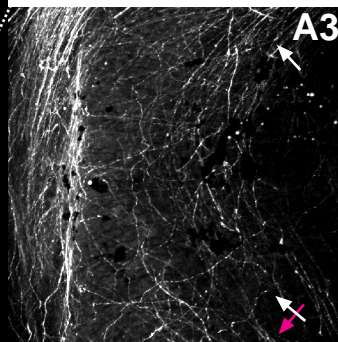

A2

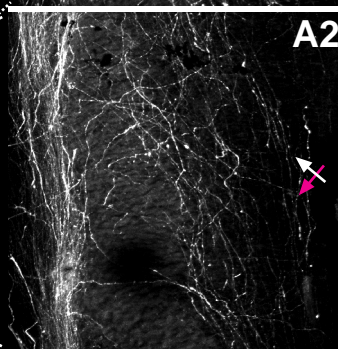

A1

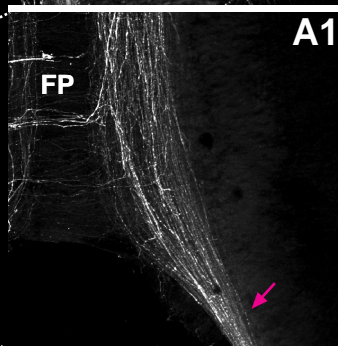

Supplement: Additional file 6 — Lhx2 + Lhx9 mediate a caudal-to-rostral change in the turning of dI2 axons. Lhx9 + GFP and Lhx2 + taumyc were expressed ectopically in dI2 neurons, utilizing the Cre/lox system and the EdI2/V1 enhancer (EdI2/V1::Cre + pCAGG-LoxP-STOP-LoxP-Lhx9-IRES-taumyc + pCAGG-LoxP-STOP-LoxP-Lhx2-IRES-taumyc). (A) At the caudal sacral level dI2Lhx2/9 axons turn caudally (A, A1). At the rostral sacral level axons turn rostrally and caudally, forming a crisscross pattern (A, A2). Rostral to the lumbar level dI2Lhx2/9 axons turn rostrally (A, A3). (B) The electroporated axons co-express GFP and taumyc. (C) A schematic illustration of the phenotype of dI2Lhx2/9axonal cues. c, cervical level; b, brachial level; FP, floor plate; l, lumbar level, s, sacral level; t, thoracic level. Scale bars: 150 μm (A, B); 75 μm (A1–A3). [file 1749-8104-4-21-S6.pdf]
